# Genome-Wide Analysis of *Escherichia coli* Isolated from Dairy Animals Identifies Virulence Factors and Genes Enriched in Multidrug-Resistant Strains

**DOI:** 10.3390/antibiotics12101559

**Published:** 2023-10-23

**Authors:** Bradd J. Haley, Seon Woo Kim, Serajus Salaheen, Ernest Hovingh, Jo Ann S. Van Kessel

**Affiliations:** 1 Environmental Microbial and Food Safety Laboratory, Beltsville Agricultural Research Center, Agricultural Research Service, United States Department of Agriculture, 307 Center Drive, Beltsville, MD 20705, USA; seonwoo.kim@usda.gov (S.W.K.);; 2 Department of Veterinary and Biomedical Sciences, Pennsylvania State University, University Park, PA 16802, USA

**Keywords:** antimicrobial resistance, multidrug resistance, *Escherichia coli*, dairy

## Abstract

The gastrointestinal tracts of dairy calves and cows are reservoirs of antimicrobial-resistant bacteria (ARB), which are present regardless of previous antimicrobial therapy. Young calves harbor a greater abundance of resistant bacteria than older cows, but the factors driving this high abundance are unknown. Here, we aimed to fully characterize the genomes of multidrug-resistant (MDR) and antimicrobial-susceptible *Escherichia coli* strains isolated from pre-weaned calves, post-weaned calves, dry cows, and lactating cows and to identify the accessory genes that are associated with the MDR genotype to discover genetic targets that can be exploited to mitigate antimicrobial resistance in dairy farms. Results indicated that both susceptible and resistant *E. coli* isolates recovered from animals on commercial dairy operations were highly diverse and encoded a large pool of virulence factors. In total, 838 transferrable antimicrobial resistance genes (ARGs) were detected, with genes conferring resistance to aminoglycosides being the most common. Multiple sequence types (STs) associated with mild to severe human gastrointestinal and extraintestinal infections were identified. A Fisher’s Exact Test identified 619 genes (ARGs and non-ARGs) that were significantly enriched in MDR isolates and 147 genes that were significantly enriched in susceptible isolates. Significantly enriched genes in MDR isolates included the iron scavenging aerobactin synthesis and receptor genes (*iucABCD*-*iutA*) and the *sitABCD* system, as well as the P fimbriae *pap* genes, *myo*-inositol catabolism (*iolABCDEG-iatA*), and ascorbate transport genes (*ulaABC*). The results of this study demonstrate a highly diverse population of *E. coli* in commercial dairy operations, some of which encode virulence genes responsible for severe human infections and resistance to antibiotics of human health significance. Further, the enriched accessory genes in MDR isolates (aerobactin, *sit*, P fimbriae, and *myo*-inositol catabolism and ascorbate transport genes) represent potential targets for reducing colonization of antimicrobial-resistant bacteria in the calf gut.

## 1. Introduction

Multidrug-resistant (MDR) *Escherichia coli* are frequently isolated from dairy cow and calf feces, unpasteurized cow’s milk, and culled dairy cow beef and are a major component of the suite of antimicrobial-resistant bacteria (ARB) carried by young calves [1,2,3]. A minority of these *E. coli* are enterohemorrhagic (EHEC) Shiga toxin-encoding strains that can cause moderate to severe gastrointestinal disease, septicemia, hemolytic uremia syndrome (HUS), chronic sequelae, and possibly death [4,5]. However, *E. coli* is highly diverse, with many suites of virulence and fitness factors (VFFs) that can cause both mild to severe gastrointestinal and extra-intestinal infections, such as urinary tract infections (UTIs) and meningitis [6]. Major non-EHEC strains causing human disease are typically classified into pathovars (pathotypes) based on the presence of different suites of virulence factors; strains that contain overlapping pathovar suites of these genes are considered hybrid pathovars. Dairy animals are known reservoirs of these MDR or antimicrobial-susceptible pathogens, but most studies of *E. coli* from these animals have either targeted only a few farms, have focused on a specific resistance phenotype/profile or pathovar, or have not utilized a genome-wide scale approach to evaluate the diversity and dynamics of these organisms in the dairy farm environment.

Dairy cow and calf feces are known reservoirs of ARGs that can contaminate milk, meat, other animals, animal handlers, and the environment [7,8]. Most studies using sensitive molecular techniques have shown that most, if not all, dairy animals carry antimicrobial-resistant bacteria (ARB) in their lower gastrointestinal system and feces, and this is consistent even in the absence of antimicrobial therapy [9,10,11,12]. Multiple studies have demonstrated that antimicrobial administration results in a transient or no increase in antimicrobial resistance (AMR) in the feces and that animals that were never exposed to these drugs may, at times, have the same level of resistance as conventionally raised animals [13,14,15]. Further, it has been repeatedly demonstrated that young calves typically carry a higher level of resistance than older lactating or dry cows, and this, too, is true in the absence of antimicrobial administration [1,2,8,16,17]. However, these calves are exposed to their dam’s microbial communities during and immediately after birth, as well as the surrounding birthing pen environment, all of which have a lower ratio of resistant to susceptible bacteria than that found in calf feces. The dam, the farm environment, colostrum, and milk/milk replacer are the sources of these ARBs, but not much is known about the genetic factors of these bacteria that influence their enrichment and persistence in the calf gut. This information could be used to potentially identify intervention targets to reduce the carriage of ARBs in dairy animals and possibly other food animals. Here, we used a genome-wide approach to evaluate the AMR profiles and virulence factor diversity of *E. coli* collected from pre-weaned calves, post-weaned calves, dry cows, and lactating cows on 80 commercial dairy farms. We also identify non-resistance conferring genes that co-occur with the MDR phenotype with the objective of identifying potential intervention targets to reduce the occurrence of MDR *E. coli* in dairy calves.

## 2. Materials and Methods

*E. coli* isolates were selected from a previously published study of antimicrobial resistance in bacteria isolated from the feces of animals in different age groups on 80 commercial dairy farms [1]. Selection was based on farm, animal group, and antibiotic sensitivity. Isolates were provisionally prescreened for antibiotic sensitivity by patch-plating onto eight petri dishes, each supplemented with a different class of antibiotics. Based on these data, one isolate per resistance group (MDR or susceptible), per animal group (pre-weaned calves, post-weaned calves, lactating cows, and dry cows), and per farm was identified. This resulted in eight groups of isolates (susceptible dry cow isolates = 315; MDR dry cow isolates = 52; susceptible lactating cow isolates = 1033; MDR lactating cow isolates = 136; susceptible post-weaned calf isolates = 221; MDR post-weaned calf isolates = 140; susceptible pre-weaned calf isolates = 132; MDR pre-weaned calf isolates = 284). Within each of these groups, random numbers were assigned to each isolate using a non-redundant random number generator in Microsoft Excel v. 16.77.1 (Microsoft Corporation, Redmond, WA, USA), and these numbers were reordered from smallest to largest, and the smallest number for each animal group within each farm was selected for genome sequencing.

Selected isolates were grown overnight at 37 °C in L broth (per 1 L: enzymatic digest of casein 10 g, yeast extract 5 g, sodium chloride 5 g), and DNA was extracted from these overnight cultures using a QiaCube platform (Qiagen, Hilden, Germany). Genome sequencing libraries were constructed using a Nextera XT kit (Illumina, La Jolla, CA, USA), and 2 × 150 bp paired-end sequencing was conducted on a NextSeq 500 platform (Illumina) with a High Output flow cell. After demultiplexing the data, reads were trimmed of adaptors, sequencing contaminants, and phiX sequences using DeconSeq [18] and then trimmed for quality and length using Trimmomatic (LEADING:20 TRAILING:20 SLIDINGWINDOW:4:20 MINLEN:36) [19]. These cleaned and curated reads were assembled using SPAdes V. 3.14.1 [20]. The genome sequencing data have been deposited at NCBI (Supplementary File S4).

Core genome SNPs were identified by aligning the 264 *E. coli* genomes used in this study with 118 publicly available *Escherichia* genomes representing the major phylogenetic groups (A, B1, B2, C, D, E, F, and G) and the near neighbors, *E. fergussoni* and *E. albertii*, downloaded from NCBI using the Harvest package [21]. ParSNP was run with the parameters -c and -x and the complete chromosome of *E. coli* K-12 substrate MG1655 (NCBI accession: NC_000913.3) as the reference genome (-r). Identified SNPs were used to infer a maximum likelihood tree with 1000 bootstrap replicates under default settings using RAxML [22].

Genome sequences were interrogated for transferrable ARGs, as well as SNPs, conferring resistance to antibiotics using the ResFinder 4.1 database with default settings (threshold for %ID = 90%, minimum length = 60%) [23]. Since our aim was to focus on ARGs, an isolate that was provisionally categorized as phenotypically susceptible but was found to carry an ARG (or ARGs associated with resistance to less than three classes of antimicrobials) was reclassified as “resistant” (R) and replaced with a phenotypically susceptible isolate from the same animal group and farm. This substituted isolate was then genomically confirmed as ARG-free. The same approach was taken to genomically validate the presence of ARGs and SNPs conferring resistance to three or more classes of antibiotics in provisionally phenotypical MDR isolates. A genome that encoded ARGs or SNPs conferring resistance to one or two classes of antibiotics was classified as “resistant” (R) and was included in that group for some downstream analyses. The final number of genome sequences used for this study was 108 multidrug-resistant (MDR), 36 antimicrobial-resistant (R), and 120 antimicrobial-susceptible (S) sequences.

Genotypic MDR genomes were identified as those genomes that encoded genes and SNPs known to confer resistance to at least three classes of antibiotics (identified as “MDR” in the analysis). Genotypic antibiotic-resistant isolates were those encoding genes or SNPs known to confer resistance to one or two classes of antibiotics (identified as “R”). Genotypic susceptible genomes were those that did not encode any known genes or SNPs that confer resistance to any antibiotics (identified as “S”). Isolates were not initially selected for sequencing based on resistance profile but were selected based on whether they were provisionally resistant to three or more antibiotics or no antibiotics, and, thus, the ARG content reflects an unbiased selection of MDR isolates. Sequence types (STs) and plasmid replicons were identified using MLST 2.0 [24,25] and PlasmidFinder 2.1 [26] with default settings. For genomes that did not match an exact ST in the MLST 2.0, the raw reads were uploaded to the Enterobase database for novel ST assignment [27].

Since virulence genes are numerous, highly diverse, and do not exclusively function to cause an infection within a mammalian host but also at times aid in survival within and outside of the host, we labeled these genes and the known major virulence factors collectively as “virulence and fitness factors” (VFF). VFFs were identified with ABRicate under default settings [28].

Identification of the pangenome was conducted by annotating the assembled genomes in PROKKA [29] under default settings and then uploading the gff3 files into Roary [30] under default settings. Using this pangenome, genes that were enriched (significantly more abundant) in MDR, as well as susceptible genomes, were identified using a Fisher’s Exact Test with the package “exact2 × 2” in R. To limit the proportion of falsely significant results, q-values (FDR—False Discovery Rate) [31] were estimated for all genes using the package “qvalue” in R (q-values are the proportion of genes that are identified as significant that are estimated to be falsely significant). A q-value threshold of <0.05 was used to identify significant features. Signal proteins were identified among the translated enriched genes to identify secreted proteins using SignalP 5.0 [32].

To visualize the distances between the accessory gene content (present in <99% of genomes) of MDR and susceptible genomes, a non-metric multidimensional scaling (NMDS) analysis using the Jaccard distance metric was inferred, followed by an analysis of similarities (ANOSIM) of the pangenomes of these two groups with and without ARGs included in the analyses using the R package “vegan”. To determine if MDR or S genomes were randomly distributed among the phylogenetic groups, a χ^2^ test was conducted in R.

## 3. Results

### 3.1. Antimicrobial Resistance Genes

In total, there were 838 ARGs detected, 46 of which were unique genes. Genomes encoded genes conferring resistance to between 0 and 8 classes of antibiotics (Table 1). The median and average number of classes to which MDR isolates were resistant were 5 and 4.6, respectively. Isolate genomes encoded between 0 and 16 ARGs, including resistance-conferring SNPs. Among the MDR isolates, the median and average numbers of ARGs per isolate were 7 and 7.3, respectively. In total, 42 resistance-conferring SNPs were detected in a total of 31 genomes. Among the genomes in which these SNPs were detected, the maximum number of SNPs detected was five and the median was 1.

There was a total of 341 aminoglycoside, 140 tetracycline, 134 sulfonamide, 115 β-lactam, 50 phenicol, 10 macrolide-lincosamide-streptogramin B (MLS), five fosfomycin, and one fluoroquinolone resistance genes detected in all the MDR and R genomes (Table 1). Colistin, fusidic acid, glycopeptide, nitroimidazole, oxazolidinone, and rifampicin resistance genes were not detected in any of the genomes. In total, there were 109 isolates that encoded aminoglycoside ARGs, and 101 of these encoded more than one aminoglycoside ARG. There were only 32, 27, and 18 isolates that encoded more than one sulfonamide, β-lactam, or tetracycline ARG, respectively. The ten most frequently detected ARGs, in order of decreasing frequency, were *aph(6)-Id* (94 isolates), *aph(3″)-Ib* (93 isolates), *sul2* (90 isolates), *tetB* (66 isolates), *bla*_CMY-2_ (65 isolates), *aph(3′)-Ia* (61 isolates), *tetA* (58 isolates), *sul1* (40 isolates), *floR* (39 isolates), *bla*_TEM-1B_ (34), and *aadA1* (22 isolates). Multiple transferrable ARGs conferring resistance or reduced susceptibility to antibiotics critical to human health were identified among the MDR isolates. These include the ESBLs *bla*_CMY-2_, *bla*_CTX-M-1_, *bla*_CTX-M-14_, *bla*_CTX-M-15_, *bla*_TEM-214_, macrolide resistance genes *mphA*, *mphB*, and the fluoroquinolone resistance gene *aac(6′)-Ib-cr*.

Point mutations in housekeeping genes that conferred resistance to antibiotics were also identified among the isolates (Table 1). Specifically, two mutation types were observed in *gyrA* (*gyrA* p.S83L and *gyrA* p.D87N), four in *parC* (*parC* p.A56T, *parC* p.S57T, *parC* p.S80I, *parC* p.E84K), one in *parE* (*parE* p.S458A), and two in the *ampC* promoter (*ampC* promoter n.-32T>A and n.-42C>T). Non-synonymous mutations in *gyrA*, *parC*, and *parE* are known to confer resistance to fluoroquinolones. Of these, the two most abundant were *gyrA* p.S83L and *parC* p.A56T, and they were identified in six isolates each. In total, 18 genomes encoded SNPs in the *ampC* promoter, and none of these encoded SNPs in *gyrA*, *parC*, or *parE*. Six isolates encoded the *gyrA* p.S83L mutation, and three of these encoded the *gyrA* p.D87N mutation. Four of the six isolates with a *gyrA* mutation also encoded mutations in *parC,* and one of these encoded mutations in *parE*. Eleven isolates encoded *parC* mutations, with six of them having *parC* p.A56T, four having *parC* p.S80I, three having *parC* p.S57T, and one having *parC* p.E84K. The latter isolate simultaneously encoded *parC* p.A56T and *parC* p.S80I. One isolate encoded *parE* p.S458A, and this isolate also encoded *gyrA* p.S83L, *gyrA* p.D87N, and *parC* p.S80I. Of the 31 isolates encoding these SNPs, 29 were the MDR genotype, one encoded one other ARG, and one encoded no other ARGs.

### 3.2. Biocide and Metal Resistance Genes

Transferrable biocide resistance genes (BRGs) and metal resistance genes (MRGs) were identified in many genomes; MDR genomes typically encoded more of these resistance genes than R or S genomes (Table 1). For each resistance category (MDR, R, and S), at least two genomes encoded copper resistance genes (*pco*), with five MDR genomes encoding this operon. Ten genomes encoded silver resistance genes (*sil*). The majority of the mercury resistance-encoding genomes were MDR (43 genomes) compared with R and S genomes (1 genome each). Similarly, the biocide resistance gene, *qacE*Δ, was found in 42 MDR genomes and 2 R genomes, but it was not found in any S genomes.

### 3.3. Co-Occurrence between ARGs, MRGs, and BRGs

**Figure 1:**
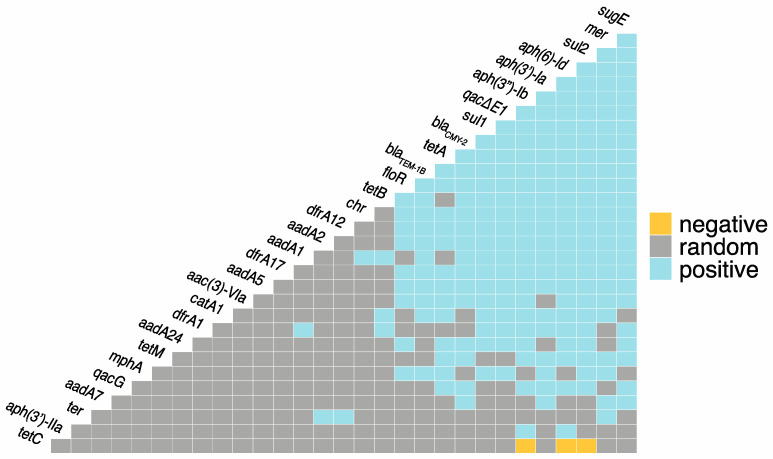
Co-occurrence among antimicrobial resistance genes (ARGs), metal resistance genes (MRGs), and biocide resistance genes (BRGs). Blue = statistically significant positive co-occurrence between genes. Yellow = statistically significant negative co-occurrence between genes. Grey = random co-occurrence.

In total, there were 222 positive co-occurrences between and among the identified ARGs, BRGs, and MRGs, and only three negative co-occurrences (Figure 1; Supplementary File S1). The most frequent co-occurrences between ARGs were *aph(3″)-Ib*-*aph(6′)-Id*, *aph(3″)-Ib*-sul2, and *aph(6′)-Id*-*sul2*. Azithromycin resistance gene, *mphA*, had positive co-occurrences with ARGs *sul1*, *sul2*, *tetA*, *bla*_TEM-1B_, and *floR*, as well as BRG *qacE*∆*1* and MRG *mer*. *bla*_CMY-2_ had positive co-occurrences with 18 other ARGs, as well as BRGs *qacE*∆*1*, *qacG*, *sugE*, and MRGs *mer* and *chr*. The most frequent co-occurrences between BRGs were *sugE*-*qacE*∆*1* and *sugE*-*qacE*∆*1*. Both *qacE*∆*1* and *sugE* had twenty positive co-occurrences with ARGs. *qacE*∆*1* had the most co-occurrences with *sul1,* and *sugE* had the most co-occurrences with *bla*_CMY-2_. The only co-occurrences between individual MRGs were *mer*-*ter* and *mer*-*chr*. Mercury resistance genes (*mer*) had 20 positive co-occurrences with ARGs as well as *sugE* and *ter*. Chromate resistance genes (*chr*) had positive co-occurrence with 10 ARGs, and tellurium resistance (*ter*) had positive co-occurrences with two ARGs, *aadA2* and *dfrA12*. The only observed negative co-occurrences were between *tetC* and *aph(3″)-Ib*, *aph(6′)-Id*, and *sul2*.

### 3.4. Plasmid Replicon Diversity

There were 708 total plasmid replicons comprising 42 different replicon types identified among the isolates (Table 2). The most frequently detected replicon was IncFIB(AP001918), which was identified in 170 isolates, followed by ColRNAI, IncFIA, IncI1_Alpha, IncFIC(FII), and IncA/C2, which were detected in 92, 87, 47, 37, and 32 genomes, respectively. For all resistance groups (MDR, R, and S), the IncFIB(AP001918) plasmid was the most frequently detected. In terms of the percentage of replicon presence, the greatest difference between MDR and S genomes was in IncFIB(AP001918), which was carried by 65% of S genomes and 40% of MDR genomes. Similarly, appreciable differences were identified in the carriage rates of IncFIC(FII), IncA/C2, and IncFIA between MDR and S genomes (19.1%, 15.8%, and 11.9% difference between MDR and S genomes).

### 3.5. Genomic Diversity

In total, 142 STs were identified among the isolates, with ST10, ST58, ST88, and ST56 being the most frequently detected, which were identified 20, 18, 10, and 7 times, respectively (Table 1). Among the MDR isolates, ST10 was the most frequently detected ST (13 isolates). Among the susceptible isolates, ST58 was the most frequently detected ST (10 isolates). Among the isolates that were resistant, except for MDR, ST95 was the most frequently detected, followed by ST10 (three and two isolates, respectively). Within the MDR isolates, ST10 was the most frequently detected ST from pre- and post-weaned calves (ST56 was detected as many times as ST10 in post-weaned calves). ST88 was the most frequently detected ST among MDR isolates from lactating cows (five isolates). ST58 was the most frequently detected ST among MDR isolates from dry cows. Within the susceptible isolates, ST58 was the most frequently detected among lactating cow isolates, while ST164 was the most frequently detected among dry cow isolates; ST32 was the most frequently detected among pre-weaned calf isolates, and ST329 and ST2521 were the most frequently detected among post-weaned calf isolates.

**Figure 2:**
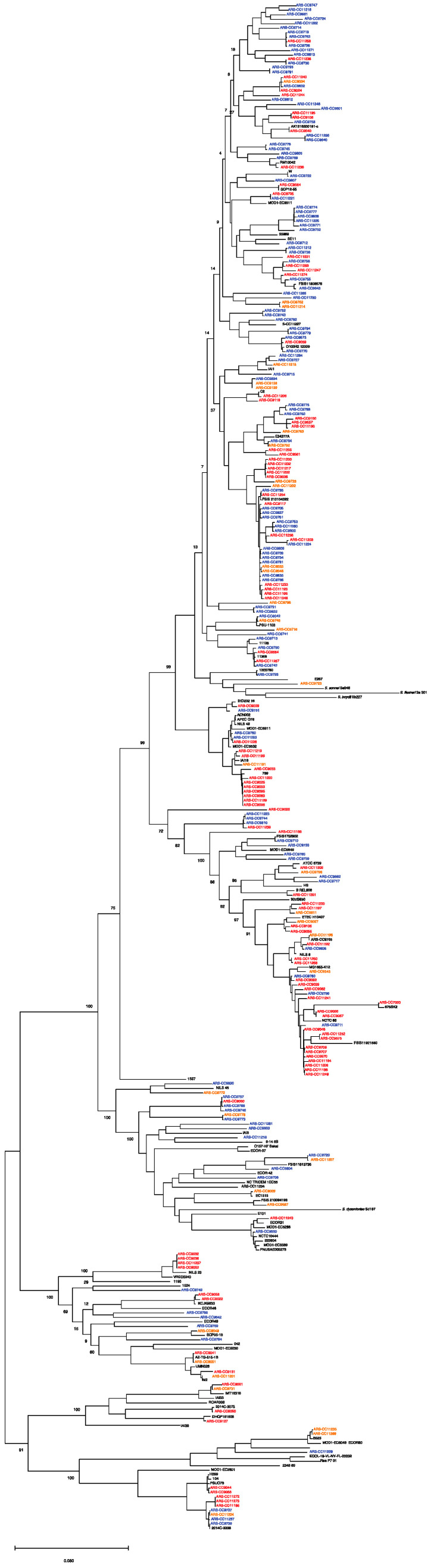
Maximum likelihood core genome phylogeny of study genomes (n = 264) and reference genomes (n = 94) of the major phylogenetic groups. The tree is rooted in *E. albertii*. Red = multidrug-resistant genomes. Orange = antimicrobial-resistant genomes. Blue = antimicrobial-susceptible genomes. The maximum likelihood tree was inferred using RaxML with 1000 bootstrap replicates. Bar represents number of substitutions per site.

**Figure 3:**
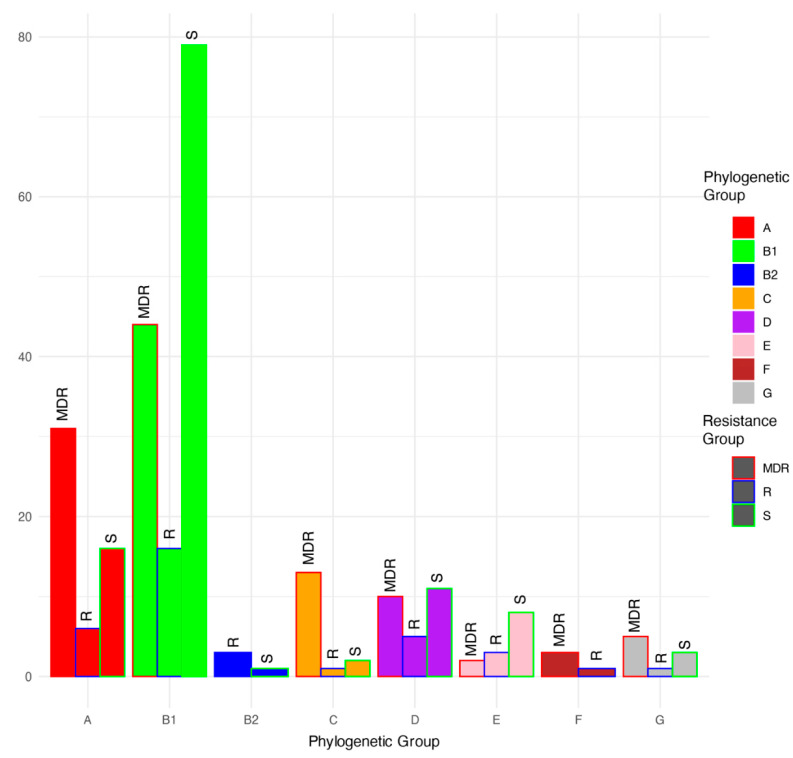
Proportions (%) of multidrug-resistant (MDR), antimicrobial-resistant (R), and antimicrobial-susceptible (S) isolates within each phylogenetic group.

Based on a phylogenetic comparison of the genomes with *Escherichia* genomes downloaded from NCBI, the isolates were classified into phylogroups A (53 isolates), B1 (139 isolates), B2 (4 isolates), C (16 isolates), D (26 isolates), E (13 isolates), F (4 isolates), and G (9 isolates) (Figure 2 and Figure 3; Table 1). Among the MDR group, isolates were classified as phylogroups B1, A, C, D, G, F, and E and were detected 44, 31, 13, 10, 5, 3, and 2 times, respectively. Among the resistant isolates, B1, A, D, E, B2, C, F, and G were detected 16, 6, 5, 3, 3, 1, 1, and 1 times, respectively. Among the susceptible isolates, B1, A, D, E, G, C, and B2 were detected 79, 16, 11, 8, 3, 2, and 1 times, respectively. MDR genomes were more likely to be phylogenetic groups A and C (residuals = 2.0 and 2.5, respectively), and S genomes were more likely to be phylogenetic group B1 (residual = 1.99; Chi-square test, χ^2^ = 46.629, *p* < 0.001).

### 3.6. Virulence and Fitness Factors

Genomes were interrogated for a broad suite of virulence and fitness factors (VFFs) that have both major and minor roles in pathogenesis, intra-host survival, and between-host transmission. In total, 49,407 VFFs were identified among the isolates, with multiple VFFs identified in all genomes (Supplementary File S2). The mean and median number of VFFs per isolate were 187 and 186, respectively. The highest number of VFFs identified in any isolate was 279, and the lowest was 114.

**Figure 4:**
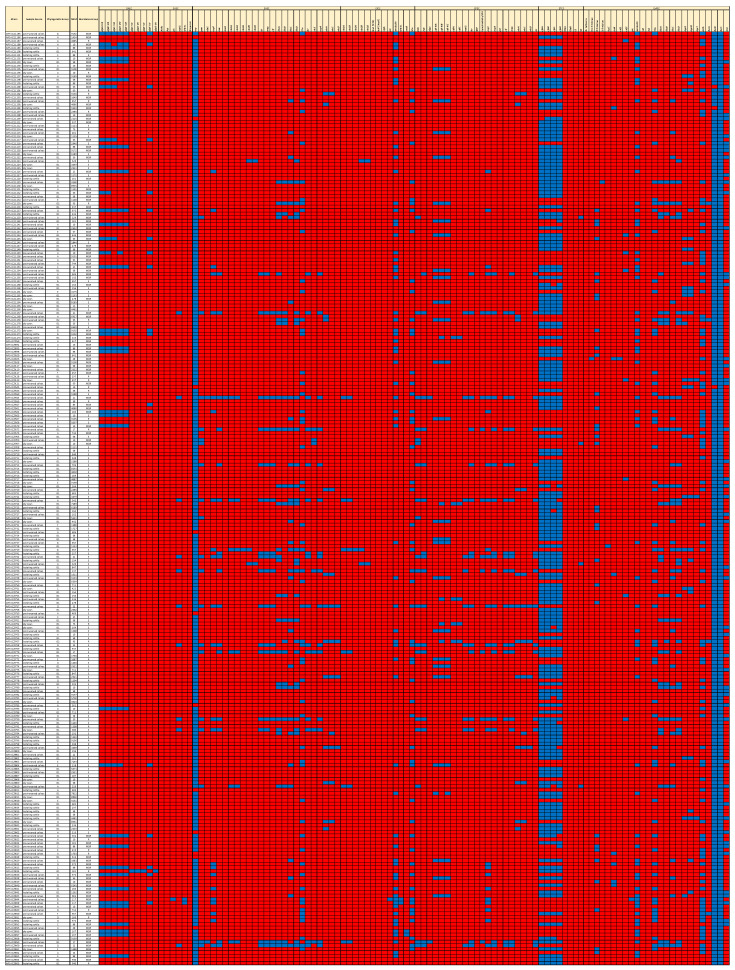
Presence/absence heatmap of virulence and fitness factors (VFFs) of the major pathovars. Blue = present. Red = absent. MLST = Multi-Locus Sequence Type.

Multiple isolates with VFFs known to play significant roles in the pathogenesis of the major pathovars were detected (Figure 4). The sequences of nine strains (3.4% of the total) significantly aligned with the Shiga toxin genes of Shiga toxigenic *E. coli* (STEC). Six of these nine genomes encoded *stx1A* and *stx1B*, one encoded *stx2A,* three encoded *stx2B*, two encoded *stx2d*, and a single genome encoded *stx1A*, *stx1B*, *stx2A*, and *stx2B*. Among the STEC genomes, four encoded the intestinal adherence factor, intimin (*eae*), which is integral in STEC pathogenesis, indicating that these isolates were potential enterohemorrhagic *E. coli* (EHEC). Two of these, O103:H2 and O45:H2, are considered adulterants by the USDA Food Safety and Inspection Service [33]. Based on the presence of *eae* in their genomes, 16 isolates were determined to be enteropathogenic *E. coli* (EPEC). Of these, two were genotypically resistant, five were MDR, and nine were susceptible. Two MDR genomes and two susceptible genomes encoded the heat-stable enterotoxin *estIa* gene (STa) of enterotoxigenic *E. coli* (ETEC), but these did not encode the LT heat-labile toxin. Four isolates were characterized as uropathogenic *E. coli* (UPEC) based on the presence of *chuA* (heme-binding protein), *fyuA* (yersiniabactin receptor), and *vat* (autotransporter serine protease toxin). Two of these were MDR ST117, and two were resistant ST95 [34]. In total, 41 isolates (15% of the total) were identified as extraintestinal pathogenic *E. coli* (ExPEC) based on the presence of at least two of the following: VFs *pap* (P fimbriae), *sfa*/*foc* (S and F1C fimbriae), *afa/draBC* (Dr binding adhesins), *kpsM* (group 2 capsule), and *iutA* (aerobactin receptor) in their genomes. Thirty-four of these isolates were MDR; three were resistant, and four were susceptible. Among these ExPEC isolates, 16 different STs were identified, with ST10, ST88, and ST973 being the most frequently detected. Multiple other ExPEC genes were detected, such as *iroN* (salmochelin siderophore receptor), *ibeABC* (invasin of brain endothelial cells), *sitABC* (iron transport protein), *cnf* (cytotoxic necrotizing factor), *cdt* (cytolethal distending toxin), and *pic* (serine protease autotransporter) (Figure 4, Supplementary File S2). In total, 60 isolates (22%) were members of STs that are among the most common causes of ExPEC infections, including ST10, ST23, ST38, ST58, ST69, ST88, ST95, ST117, and ST167.

### 3.7. Accessory Genes Associated with the MDR and Susceptible Genotypes

**Figure 5:**
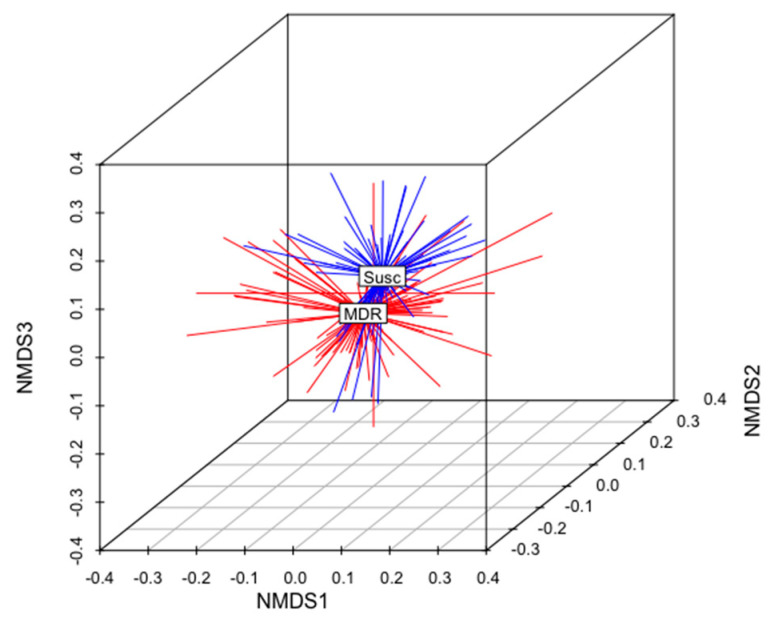
Non-metric multidimensional scaling (NMDS) analysis of the presence and absence of genes in the genomes of all MDR and susceptible isolates in this analysis (k = 3; stress = 0.17; ANOSIM R = 0.16; *p* < 0.01). The centroid of each group (MDR or Susc) is labeled. MDR genomes are shown in red. S genomes are shown in blue.

**Figure 6:**
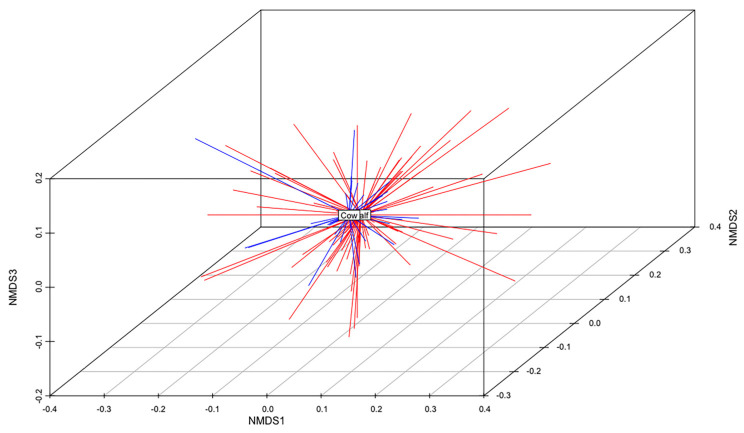
Non-metric multidimensional scaling (NMDS) analysis of the presence and absence of genes in the genomes of all MDR isolates from calves and adult cows (k = 3; stress = 0.15; ANOSIM R = −0.08; *p* = 0.972). The centroid of each group (MDR calf or MDR cow) is labeled. MDR calf genomes are shown in red. MDR cow genomes are shown in blue.

The total gene content of the MDR and S genomes were compared to identify differences in accessory genes between these two groups of isolates. A non-metric multidimensional scaling (NMDS) analysis and an analysis of similarities (anosim) test demonstrated that the gene content of MDR genomes was different than that of S genomes (Figure 5). When all ARGs were removed from this analysis, this relationship remained statistically significant (anosim R = 0.16, *p* < 0.001), indicating that MDR genomes and susceptible genomes were slightly, yet significantly, different from each other, and these differences were not due to the presence of ARGs (Figure 5). There was no difference in the gene content of MDR *E. coli* isolated from calves than those isolated from adult cows (anosim *p* = 0.972), indicating that the gene contents of MDR strains shed by cows were not significantly different than those shed by calves (Figure 6).

**Figure 7:**
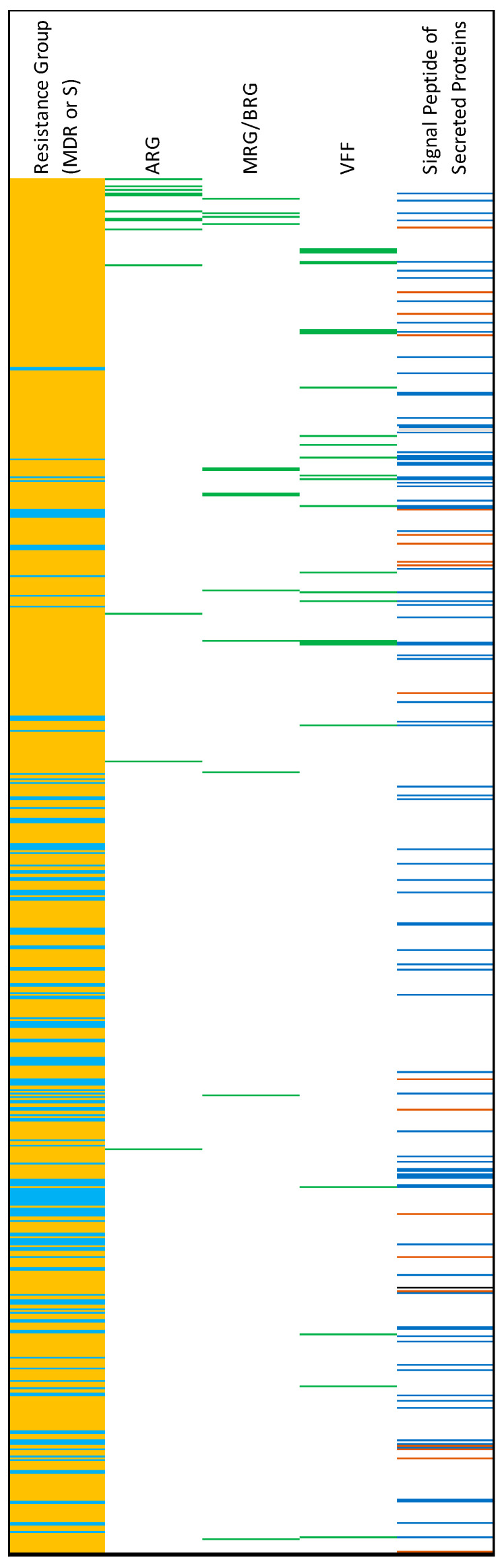
Results of the Fisher’s Exact Test analysis of gene enrichment in MDR and susceptible (S) genomes. Light-blue on far-left column = genes enriched in S genomes. Orange = genes enriched in MDR genomes. For columns from 2 to 4: green = the corresponding gene is an antimicrobial resistance gene (ARG), metal resistance gene/biocide resistance gene (MRG/BRG), or virulence and fitness factor (VFF). For the last column, prediction of signal peptides of secreted proteins: blue = SP(Sec/SPI), brown = LIPO(Sec/SPII), black = TAT(Tat/SPI).

There was a total of 766 accessory genes that were significantly enriched in either MDR strains or in susceptible strains (Fisher’s Exact Test, q < 0.05) (Figure 7; Supplementary File S3). Of these, 619 (80%) were enriched in MDR strains, and 147 were enriched in susceptible strains. Thirteen of the 619 accessory genes that were enriched in MDR strains were identified as ARGs. Signal proteins (markers of secreted proteins) were detected in 86 protein products of the enriched genes in MDR strains (Figure 7; Supplementary File S3). When the enriched genes in the susceptible strains were translated, 22 signal proteins were detected.

VFFs were also identified among the accessory genes enriched in either MDR or S genomes (Supplementary File S3). In the S genomes, these included *cfaC* (Mat/Ecp fimbriae outer membrane usher protein), *cfaB* (fimbrial protein), *fimC* (fimbrial chaperone protein), and *fimI* (long polar fimbria major subunit LpfA). In the MDR genomes, these included *afa* (adhesin), *f17c-a* (major type 1 subunit fimbrin), *f17d-C* (outer membrane usher protein), *iuc-iutA* (aerobactin synthesis and receptor), *orgA* (type III secretion apparatus protein OrgA/MxiK), *pap* (P-fimbriae), *sit* (iron and manganese transport), *tia* (Tia invasion determinant), and *yjaa* (YjaA family stress response protein).

Of the 619 genes enriched in MDR strains, 238 (38%) were annotated as known protein-coding genes. When assigned to KEGG functional categories, 26.8% were assigned to the signaling and cellular processes category of the protein families; 14.7% were assigned to the environmental information processing category; 13.4% were assigned to the unclassified genetic information processing category; 11.7% were assigned to the genetic information processing category of the protein families; 8.8% were assigned to the carbohydrate metabolism category, and 4.2% were assigned to the unclassified metabolism category.

Notable genes of interest known to confer significant phenotypes on cells that encode them that were enriched in the MDR genome include multiple iron acquisition systems, such as *iucABCD-iutA* (aerobactin synthesis and receptor), *fecABCD* (iron transport operon), *sitACD* (involved in iron and manganese transport), *papABCD* (P fimbriae), *myo*-inositol catabolism and transport genes *iolABCDEG-iatA*, and ascorbate metabolism genes *ulaABC* (Table 3). Multiple metal and biocide resistance genes were also enriched in MDR genomes. These include resistance to mercury (*mer*), copper (*pco*), silver (*sil*), and quaternary ammonia compounds (*qacE*Δ*1*) (Supplementary File S3).

## 4. Discussion

Results of this study demonstrate that genotypically antimicrobial-resistant and virulent *E. coli* are shed by dairy animals of all ages. Genes integral in the infection processes of all the major pathovars were detected among the strains. In previous studies, non-STEC strains isolated from bovine sources were frequently considered non-type-specific *E. coli*. However, a deeper genomic investigation of the strains in this study demonstrates that they often encode multiple VFFs as well as ARGs, demonstrating their human and animal health significance. Among these *E. coli*, STEC adulterant and ExPEC strains were repeatedly isolated. ExPEC strains are frequently MDR [35], and a high percentage of MDR strains in this study encoded ExPEC VFs, more so than the integral VFs of other pathovars.

Multiple ARGs conferring resistance to antimicrobials of human significance, particularly β-lactamases (*bla*_CMY_ and *bla*_CTX-M_) and azithromycin (macrolide) resistance genes (*mphA* and *mphB*), along with point mutations conferring resistance to ciprofloxacin were identified in the genomes of *E. coli* isolated from the feces of dairy animals. β-lactams are among the most frequently used antibiotics in human clinical medicines, and resistance to these therapeutics has been increasing globally [36,37]. Fieldwork on these study farms demonstrated the presence of *E. coli* resistant to these antibiotics in the majority of farms, indicating that they are prevalent in dairy systems in the geographic study region [1]. Interestingly, *bla*_CTX-M_ and, in particular, *bla*_CMY_ were identified in more than half of MDR isolates, indicating that they were a significant component of MDR *E. coli* in these environments. This is further evidenced by the frequent co-occurrence of *bla*_CMY_ with other ARGs in the *E. coli* genomes.

Based on an analysis of *E. coli* and the resistomes of calf feces, a higher ratio of resistant to susceptible bacteria is present in pre-weaned calf feces than in the feces of older animals [1,16]. Cao et al. [1] demonstrated this high level of resistance in pre- and post-weaned calves compared to dry or lactating cows, with pre-weaned calf feces harboring more resistant *E. coli* and more MDR *E. coli* than post-weaned calf feces. Previous studies have indicated that ARBs from teat surfaces can contaminate colostrum, which might seed the calf gut with ARBs, and that changes in diet may be, in part, responsible for the decrease in resistance abundance in these young animals. However, calves fed colostrum replacer and milk replacer similarly harbor high levels of resistance [38] that decrease over time, suggesting that there might be a selective pressure for ARBs in the calf gut that results in relatively high abundance of these organisms in young animals.

Previous work has shown that the presence of ARGs is associated with the presence of biocide and metal resistance genes (BRGs and MRGs) and that they are often collocated on the same plasmids or mobile elements [39,40]. It has been postulated that the presence of trace metals in feed selects ARBs that have MRGs encoded in their genomes [41,42]. Similar associations between ARGs, MRGs, and BRGs were identified in this study, most notably between the MDR genotype and the mercury resistance operon and the BRG, *sugE1*. However, the results of this study further demonstrate that *E. coli* with the MDR genotype is more likely to encode multiple suites of non-MRG/non-BRG genes encoding traits that may select for these organisms even in the absence of antimicrobial therapy or trace metal/biocide exposure and that these genes may point toward potential intervention strategies to reduce the carriage of ARBs in the herd or individual animal groups.

Our analyses indicated that the accessory gene contents of MDR isolates were somewhat different from those of the susceptible isolates, even when ARGs were removed from the analyses. Further, the gene content of MDR isolates from calves was not significantly different from that of adult cows, although the ratio of resistant to susceptible isolates is higher in calves than in cows [1]. Previous work has demonstrated that calves and adult cows can harbor the same MDR *E. coli* [43]. These data indicate that there are some genes that are more abundant in MDR strains than susceptible strains and that the gene contents of these MDR strains isolated from calves are approximately similar to those isolated from adult cows. Several of these genes compose operons that are involved in metabolic mechanisms that may be involved in this high MDR *E. coli* carriage in the calf gut. Of particular interest is the high number of genes enriched in the MDR isolates that are involved in iron acquisition. Iron is a limiting nutrient that is integral in many essential cellular processes. In human pathogenic *E. coli*, iron acquisition mechanisms are frequently found in ExPEC isolates, which are commensal in the gut but potentially pathogenic in the extraintestinal environment [44]. These systems are required for ExPEC colonization outside of the human gut, and studies have shown that they are upregulated in extraintestinal environments where available iron is limited [45,46,47]. However, the possible roles of iron acquisition mechanisms in the non-human mammalian gut have not been resolved. Iron scavenging via siderophores chelates iron, which transports these compounds back to the cell. Many taxa encode siderophores in the core chromosome, as well as accessory siderophores such as the sit system, aerobactin production, and salmochelin. The calf gut environment is relatively iron-depleted, and the main source of nutrition is usually colostrum for the first two days, followed by milk, both of which have a low iron content compared to that of the diets of older animals, which consume mostly forages and grains [48]. We hypothesize that the presence of siderophore systems (*iucABCD-iutA* and *sitABCD*), which are expressed in low-iron environments and are enriched in MDR strains in dairy animals, may allow for these strains to outcompete susceptible strains that typically lack these accessory iron scavenging systems. Siderophores are expressed in low-iron environments as a competitive mechanism to acquire this essential nutrient [49,50,51,52]. In *E. coli*, these genes (*iucABCD-iutA* and *sitABCD*) are typically encoded on incF plasmids, which also frequently encode ARGs, BRGs, and MRGs. We hypothesize that the collocation of plasmid-borne siderophores and ARGs selects for the latter in the low iron environment of the calf gut. Similar associations have been shown in *E. coli* collected from veal calves, which are typically fed an iron-limited diet [53].

The presence of other genes not involved in iron scavenging that are enriched in MDR strains suggests that the selection of ARGs within the calf gut may be multifactorial. These genes include *myo*-inositol transport and catabolism genes. Inositol is a carbocyclic sugar that has multiple structural and signaling roles in both eukaryotes and prokaryotes. It is found in both cow’s milk and feeds, such as cereal grains in its phosphorylated form, inositol hexakisphosphate (InsP6), or phytate, while forages have considerably lower InsP6 concentrations. Homologs of these ORFs have been identified in *S. enterica,* which can utilize inositol as a sole carbon source in vitro, indicating that it has an influence on the survival and, possibly, persistence of strains encoding these genes in environments with available inositol [54]. The introduction of forages and grains as a “calf starter” diet roughly coincides with a decrease in the carriage of MDR strains at around two weeks, which continues through the post-weaning period, suggesting that changes in diet may alter the abundance of resistance in the gut [2]. However, these genes are typically located on the *E. coli* chromosome, while most ARGs harbored by *E. coli* are plasmid-borne. It is not clear if there is a synergistic relationship between chromosomal-encoded genes and plasmid-borne ARGs; this association needs further evaluation.

Genes representing other, less defined mechanisms were also enriched in the MDR isolates; they may also confer a selective advantage for these bacteria in the calf gut and, hence, may also represent potential intervention targets for resistance in all animal groups. A significant number of genes were annotated as hypothetical proteins, indicating that their functions are not yet known. It may be that some of these are non-functional, but multiple hypothetical proteins were predicted to be translocated across the cell membrane, which suggests that they may be involved in interactions with the extracellular environment, which could include the bovine gastrointestinal tract. Further work identifying the functions of these genes in vitro and in vivo would need to be conducted to determine if they play any significant role in the survival of MDR strains in the animal gut.

MDR *E. coli* are frequently shed from calves and cows throughout the duration of their lives. The abundance of these resistant bacteria typically peaks at around two to three weeks of life, but it is still relatively high after weaning. The first two to three weeks after birth coincides with the time during which calves are primarily fed a milk diet, after which consumption of a calf starter, which has a higher concentration of iron than milk, occurs more frequently as the calf ages. These results demonstrate a need to further evaluate the role of iron availability within the calf gut as a potential driver of the high abundance of AMR in the feces of young calves, which may reveal a relatively simple approach to reducing the carriage of MDR *E. coli* in the dairy calf gut. Such an approach could have One Health implications by mitigating or disrupting the flow of multidrug-resistant *E. coli* between dairy farms, animals, and the environment.

## Figures and Tables

**Table 1:** Isolation source, phylogenetic group, multi-locus sequence type (MLST), antimicrobial resistance genes (ARGs) separated by antimicrobial class, antimicrobial resistance-conferring point mutations, metal resistance genes (MRGs), and biocide resistance genes (BRGs) identified among the study isolates. Ami = aminoglycosides, β-Lac = β-Lactams, Flu = fluoroquinolones, Fos = Fosfomycin, MLS = macrolide-lincosamide-streptogramin B, Phe = phenicols, Sul = sulfonamide, Tet = tetracyclines, Tri = trimethoprim. As = arsenic, Cu = copper, Hg = mercury, Ag = silver, Te = Tellurium. BRGs = Biocide resistance genes. QAC = quaternary ammonium compounds.

Isolate ID	Sample Source	Phylogenetic Group	MLST	Antimicrobial Resistance Genes (ARGs)									Resistance Conferring Point Mutations				Metal Resistance Genes (MRGs)						BRGs	
				Ami	β-Lac	Flu	Fos	MLS	Phe	Sul	Tet	Tri	*gyrA*	*parC*	*parE*	*ampC*	As	Cu	Hg	Ag	Te	Chromate	sugE1	QAC
ARS-CC11185	Postweaned calves	G	9192	*aadA1*, *aph(3′)-Ia*, *aadA2*, *aph(6)-Id*, *aph(3″)-Ib*	*bla*_CMY-2_				*floR*	*sul1*, *sul2*	*tetB*, *tetA*	*dfrA1*, *dfra12*				*ampC* promoter n.-42C>T			*merA*, *merT*, *merR*, *merP*, *merC*, *merD*, *merE*			*chrA*	*sugE1*	*qacE*∆*1*
ARS-CC11186	Post-weaned calves	A	1434	*aph(3′)-Ia*, *aadA5*, *aph(6)-Id*, *aph(3″)-Ib*	*bla*_CMY-2_, *bla*_TEM-1B_				*floR*	*sul1*, *sul2*	*tetA*	*dfrA17*		*parC* p.A56T				*pcoA*, *pcoB*, *pcoC*, *pcoD*, *pcoE*, *pcoR*, *pcoS*		*silA*, *silB*, *silC*, *silP*, *silR*		*chrA*	*sugE1*	*qacE*∆*1*
ARS-CC11187	Pre-weaned calves	A	4085	*aadA1*						*sul1*						*ampC* promoter n.-42C>T			*merA*, *merT*, *merR*, *merP*, *merC*, *merD*, *merE*					*qacE*∆*1*
ARS-CC11188	post-weaned calves	A	10	*aph(3′)-Ia*, *aph(6)-Id*, *aph(3″)-Ib*	*bla*_TEM-1B_, *bla*_CMY-2_					*sul2*	*tetB*												*sugE1*	
ARS-CC11189	lactating cattle	C	88	*aph(6)-Id*, *aph(3′)-Ia*, *aph(3″)-Ib*						*sul2*	*tetB*					*ampC* promoter n.-42C>T								
ARS-CC11190	lactating cattle	B1	641	*aph(3′)-Ia*	*bla*_CMY-2_						*tetA*							*pcoA*, *pcoB*, *pcoC*, *pcoD*, *pcoE*, *pcoR*, *pcoS*		*silA*, *silB*, *silC*, *silP*, *silR*				
ARS-CC11191	lactating cattle	C	88		*bla*_CMY-2_																		*sugE1*	
ARS-CC11192	pre-weaned calves	A	10	*aph(3′)-Ia*, *aph(6)-Id*, *aph(3″)-Ib*	*bla*_CMY-2_					*sul2*	*tetB*							*pcoA*, *pcoB*, *pcoC*, *pcoD*, *pcoE*, *pcoR*, *pcoS*		*silA*, *silC*, *silP*, *silR*				
ARS-CC11193	dry cows	B1	58	*aph(3′)-Ia*, *aph(6)-Id*, *aadA24*, *aac(3)-Via*, *aph(3″)-Ib*	*bla*_CMY-2_, *bla*_TEM-1B_				*floR*	*sul1*, *sul2*	*tetB*, *tetA*								*merA*, *merT*, *merR*, *merP*, *merC*, *merD*, *merE*				*sugE1*	*qacE*∆*1*
ARS-CC11194	lactating cattle	A	10	*aph(3′)-Ia*, *aph(6)-Id*, *aph(3″)-Ib*	*bla*_CMY-2_			*mphA*	*floR*	*sul2*	*tetA*								*merA*, *merT*, *merR*, *merP*, *merC*, *merD*, *merE*			*chrA*	*sugE1*	*qacG*
ARS-CC11195	post-weaned calves	B1	9190	*aph(3″)-Ib*, *aph(6)-Id*, *aadA2*	*bla*_CMY-2_				*floR*	*sul1*, *sul2*	*tetA*	*dfrA12*							*merA*, *merT*, *merR*, *merP*, *merC*, *merD*, *merE*			*chrA*	*sugE1*	*qacE*∆*1*
ARS-CC11196	dry cows	A	10		*bla*_CTX-M1_																			
ARS-CC11197	lactating cattle	A	9189	*aph(3″)-Ib*, *aph(6)-Id*, *aadA2*	*bla*_CMY-2_				*floR*	*sul1*, *sul2*	*tetA*	*dfrA12*		*parC* p.A56T					*merA*, *merT*, *merR*, *merP*, *merC*, *merD*, *merE*			*chrA*	*sugE1*	*qacE*∆*1*
ARS-CC11198	pre-weaned calves	B1	58	*aph(6)-Id aph(3″)-Ib*	*bla*_CMY-2_, *bla*_TEM-1B_					*sul2*		*dfrA5*							*merA*, *merT*, *merR*, *merP*, *merC*, *merD*, *merE*				*sugE1*	
ARS-CC11199	lactating cattle	C	88	*aac(3)-Via*, *aadA24*, *aph(6)-Id*, *aph(3″)-Ib*	*bla*_CMY-2_, *bla*_TEM-1B_				*floR*	*sul2*	*tetA*								*merA*, *merT*, *merR*, *merP*, *merC*, *merD*, *merE*				*sugE1*	
ARS-CC11200	post-weaned calves	B1	56	*aph(3″)-Ib*, *aph(3′)-Ia*, *aph(6)-Id*, *aadA2*	*bla*_CMY-2_			*mphA*	*floR*	*sul1*, *sul2*	*tetA*, *tetB*, *tetM*	*dfrA12*							*merA*, *merT*, *merR*, *merP*, *merC*, *merD*, *merE*			*chrA*	*sugE1*	*qacE*∆*1*, *qacG*
ARS-CC11201	dry cows	D	69		*bla*_CMY-2_												*arsA*, *arsB*, *arsC*, *arsD*						*sugE1*	
ARS-CC11202	lactating cattle	B1	9194		*bla*_CMY-2_												*arsA*, *arsB*, *arsC*, *arsD*						*sugE1*	
ARS-CC11203	pre-weaned calves	B1	1049		*bla*_CTX-M1_					*sul2*	*tetA*													
ARS-CC11204	post-weaned calves	G	657		*bla*_CMY-2_																		*sugE1*	
ARS-CC11205	dry cows	B1	4086	*aph(6)-Id*, *aph(3″)-Ib*	*bla*_CMY-2_				*floR*	*sul2*	*tetA*								*merA*, *merT*, *merR*, *merP*, *merC*, *merD*, *merE*				*sugE1*	
ARS-CC11206	lactating cattle	B1	9203	*aph(3″)-Ib*, *aph(6)-Id*, *aadA1*, *aac(3)-Via*, *aadA5*					*floR*	*sul1*, *sul2*	*tetA*	*dfrA17*										*chrA*		*qacE*∆*1*
ARS-CC11207	post-weaned calves	D	2485		*bla*_CMY-2_						*tetA*												*sugE1*	
ARS-CC11208	post-weaned calves	A	10	*aac(3)-Via*, *aph(3″)-Ib*, *aph(6)-Id*	*bla*_CMY-2_				*floR*	*sul2*	*tetA*								*merT*				*sugE1*	
ARS-CC11209	pre-weaned calves	A	2325	*aph(6)-Id*, *aadA1*, *aph(3′)-Ia*, *aph(3″)-Ib*	*bla*_CMY-2_, *bla*_TEM-1B_					*sul1*	*tetB*								*merA*, *merT*, *merR*, *merP*, *merC*, *merD*				*sugE1*	*qacE*∆*1*
ARS-CC11211	dry cows	B1	937	*aph(6)-Id*, *aph(3″)-Ib*						*sul2*	*tetB*													
ARS-CC11212	post-weaned calves	B1	1123																					
ARS-CC11214	pre-weaned calves	B1	75				*fosA7.5*																	
ARS-CC11215	post-weaned calves	B1	201	*aph(3′)-Ia*																				
ARS-CC11216	dry cows	B1	1125																					
ARS-CC11217	post-weaned calves	B1	56	*aph(6)-Id*, *aph(3″)-Ib*	*bla*_TEM-1B_					*sul2*	*tetB*													
ARS-CC11218	pre-weaned calves	D	2946																					
ARS-CC11219	pre-weaned calves	C	88	*aph(3′)-Ia*, *aph(6)-Id*, *aph(3″)-Ib*						*sul2*	*tetB*					*ampC* promoter n.-42C>T								
ARS-CC11220	post-weaned calves	C	9172	*aph(3″)-Ib*, *aph(6)-Id*, *aph(3′)-Ia*					*floR*	*sul2*	*tetB*					*ampC* promoter n.-32T>A								
ARS-CC11221	dry cows	B1	6189																					
ARS-CC11222	pre-weaned calves	B1	56	*aadA2*, *aph(3′)-Ia*, *aph(3″)-Ib*, *aph(6)-Id*	*bla*_TEM-1B_					*sul1*, *sul2*	*tetA*	*dfrA12*							*merA*, *merT*, *merR*, *merP*, *merC*, *merD*, *merE*		*terY2*, *terY1*, *terW*, *terZ*, *terA*, *terB*, *terC*, *terD*, *terE*, *terF*			*qacE*∆*1*
ARS-CC11223	post-weaned calves	A	329																					
ARS-CC11224	dry cows	B1	1049																					
ARS-CC11225	dry cows	B1	2521																					
ARS-CC11226	pre-weaned calves	C	23	*aadA1*, *aph(3′)-Iia*, *aph(6)-Ic*, *aph(6)-Id*, *aph(3″)-Ib*						*sul2*	*tetB*	*dfrA1*				*ampC* promoter n.-42C>T					*terW*, *terZ*, *terA*, *terB*, *terC*, *terD*, *terE*, *terF*			
ARS-CC11227	post-weaned calves	B1	1172																					
ARS-CC11228	lactating cattle	B1	101	*aph(6)-Id*, *aph(3″)-Ib*						*sul2*	*tetB*													
ARS-CC11229	pre-weaned calves	B2	4260																					
ARS-CC11230	dry cows	A	8935																					
ARS-CC11231	lactating cattle	A	1101	*aph(6)-Id*, *aph(3″)-Ib*						*sul2*	*tetB*													
ARS-CC11232	lactating cattle	B1	56	*aph(6)-Id*, *aph(3″)-Ib*						*sul2*	*tetB*													
ARS-CC11233	pre-weaned calves	B1	58	*aac(3)-Iid*, *aadA5*	*bla*_TEM-1B_					*sul1*, *sul2*	*tetB*	*dfrA17*										*chrA*		*qacE*∆*1*
ARS-CC11234	post-weaned calves	E	1140	*aph(6)-Id*, *aph(3″)-Ib*	*bla*_CMY-2_				*floR*	*sul2*	*tetA*								*merA*, *merT*, *merR*, *merP*, *merC*, *merD*, *merE*				*sugE1*	
ARS-CC11235	dry cows	B2	95								*tetC*													
ARS-CC11236	lactating cattle	B1	937	*aph(6)-Id*, *aph(3″)-Ib*						*sul2*	*tetB*													
ARS-CC11237	pre-weaned calves	D	973	*aadA7*, *aph(3′)-Ia*, *aph(6)-Id*, *aph(3″)-Ib*	*bla*_CMY-2_					*sul1*, *sul2*									*merA*, *merT*, *merR*, *merP*, *merC*, *merD*, *merE*				*sugE1*	*qacE*∆*1*
ARS-CC11238	lactating cattle	B1	442	*aph(3″)-Ib*,*aph(6)-Id*					*floR*	*sul2*	*tetA*													
ARS-CC11239	post-weaned calves	A	329	*aph(3′)-Ia*, *aph(3″)-Ib*, *aph(6)-Id*						*sul2*	*tetB*										*terW*, *terZ*, *terA*, *terB*, *terC*, *terD*, *terE*, *terF*			
ARS-CC11240	post-weaned calves	B1	101	*aph(3′)-Ia*, *aph(6)-Id*, *aph(3″)-Ib*						*sul2*	*tetB*													
ARS-CC11241	pre-weaned calves	A	10	*aph(3′)-Ia*, *aph(6)-Id*, *aph(3″)-Ib*	*bla*_CMY-2_					*sul2*	*tetB*												*sugE1*	
ARS-CC11242	post-weaned calves	B1	2522	*aph(6)-Id*, *aph(3″)-Ib*						*sul2*	*tetB*													
ARS-CC11243	pre-weaned calves	E	57	*aph(3′)-Ia*, *aadA5*, *aph(6)-Id*, *aac(3)-Iia*, *aadA1*, *rmtE*, *aph(3″)-Ib*	*bla*_CMY-2_			*mphB*	*catA1*	*sul2*, *sul1*	*tetM*, *tetA*	*dfrA1*, *dfrA17*	*gyrA* p.S83L						*merA*, *merT*, *merR*, *merP*, *merC*, *merD*, *merE*			*chrA*	*sugE1*	*qacE*∆*1*
ARS-CC11244	post-weaned calves	B1	446	*aph(3′)-Ia*, *aadA2*, *aph(6)-Id*, *aph(3″)-Ib*	*bla*_TEM-1A_					*sul1*	*tetA*								*merA*, *merC*, *merD*, *merE*					*qacE*∆*1*
ARS-CC11245	dry cows	B1	56	*aph(6)-Id*, *aph(3″)-Ib*						*sul2*	*tetB*													
ARS-CC11246	post-weaned calves	B1	1844																					
ARS-CC11247	post-weaned calves	B1	278	*aph(3″)-Ib*, *aph(6)-Id*					*floR*	*sul2*	*tetA*													
ARS-CC11248	lactating cattle	B1	58	*aph(3′)-Ia*, *aph(6)-Id*, *aph(3″)-Ib*						*sul2*	*tetB*													
ARS-CC11249	pre-weaned calves	A	10	*aph(3′)-Ia*, *aph(6)-Id*, *aph(3″)-Ib*	*bla*_CMY-2_, *bla*_TEM-1B_					*sul2*	*tetB*												*sugE1*	
ARS-CC11250	pre-weaned calves	A	9191	*aph(3′)-Ia*, *aph(6)-Id*, *aph(3″)-Ib*	*bla*_CMY-2_						*tetB*												*sugE1*	
ARS-CC11251	pre-weaned calves	A	93	*aph(3″)-Ib*, *aph(6)-Id*, *aadA1*						*sul1*,*sul2*	*tetB*	*dfrA1*	*gyrA* p.S83L						*merA*, *merT*, *merR*, *merP*, *merC*, *merD*, *merE*					*qacE*∆*1*
ARS-CC11252	post-weaned calves	A	744	*aph(6)-Id*, *aph(3″)-Ib*	*bla*_TEM-1B_			*mphA*	*catA1*	*sul2*	*tetB*	*dfrA17*	*gyrA* p.S83L, *gyrA* p.D87N	*parC* p.A56T, *parC* p.S80I, *parC* p.E84K					*merA*, *merT*, *merR*, *merP*, *merC*, *merD*, *merE*					
ARS-CC11253	pre-weaned calves	C	23	*aph(3″)-Ib*, *aph(6)-Id*, *aph(3′)-Ia*, *aadA1*						*sul2*	*tetB*	*dfrA1*				*ampC* promoter n.-42C>T					*terW*, *terZ*, *terA*, *terB*, *terC*, *terD*, *terE*, *terF*			
ARS-CC11254	post-weaned calves	B1	58	*aph(6)-Id*, *aph(3″)-Ib*						*sul2*	*tetB*													
ARS-CC11255	post-weaned calves	A	206	*aph(3′)-Ia*, *aph(3″)-Ib*, *aph(6)-Id*, *aadA2*	*bla*_TEM-1B_				*catA1*, *floR*	*sul1*, *sul2*	*tetA*	*dfrA12*		*parC* p.A56T					*merA*, *merT*, *merR*, *merP*, *merC*, *merD*, *merE*		*terY2*, *terY1*, *terW*, *terZ*, *terA*, *terB*, *terC*, *terD*, *terE*, *terF*			*qacE*∆*1*
ARS-CC11256	post-weaned calves	B1	155	*aac(3)-Via*, *aadA24*					*floR*	*sul1*	*tetA*													*qacE*∆*1*
ARS-CC11257	pre-weaned calves	G	657																					
ARS-CC11258	lactating cattle	B1	164	*aph(6)-Id*, *aph(3″)-Ib*						*sul2*	*tetB*													
ARS-CC11260	post-weaned calves	B1	155																					
ARS-CC11261	dry cows	E	4175																					
ARS-CC11262	dry cows	B1	2163																					
ARS-CC11263	dry cows	B1	278	*aph(6)-Id*, *aph(3″)-Ib*	*bla*_TEM-1A_				*floR*	*sul2*	*tetA*													
ARS-CC11264	pre-weaned calves	B1	8185																					
ARS-CC11265	dry cows	B1	13																					
ARS-CC11266	dry cows	B1	4481																					
ARS-CC11267	pre-weaned calves	B1	21	*aac(3)-Via*, *aph(3″)-Ib*, *aph(6)-Id*	*bla*_CMY-2_				*floR*	*sul2*	*tetA*								*merA*, *merT*, *merR*, *merP*, *merC*, *merD*, *merE*		*terW*, *terZ*, *terA*, *terB*, *terC*, *terD*, *terE*, *terF*		*sugE1*	
ARS-CC11268	post-weaned calves	A	6927	*aadA24*, *aac(3)-Via*, *aph(3″)-Ib*, *aph(6)-Id*	*bla*_CMY-2_					*sul1*, *sul2*	*tetA*								*merA*, *merT*, *merR*, *merP*, *merC*, *merE*				*sugE1*	*qacE*∆*1*
ARS-CC11269	post-weaned calves	B2	95								*tetC*													
ARS-CC11270	dry cows	B1	56																					
ARS-CC11271	pre-weaned calves	B1	6559																					
ARS-CC11272	dry cows	G	9192	*aph(6)-Id*, *aph(3′)-Ia*, *aph(3″)-Ib*, *aadA1*						*sul2*	*tetB*	*dfrA1*				*ampC* promoter n.-42C>T								
ARS-CC11273	lactating cattle	G	9192	*aph(6)-Id*, *aph(3′)-Ia*, *aph(3″)-Ib*, *aadA1*						*sul2*	*tetB*	*dfrA1*				*ampC* promoter n.-42C>T								
ARS-CC11274	lactating cattle	B1	316	*aph(6)-Id*, *aph(3″)-Ib*						*sul2*	*tetB*													
ARS-CC7050	lactating cattle	A	617	*aadA5*, *aac(6′)-Ib-cr*	*bla*_CTX-M-15_	*aac(6′)-Ib-cr*		*mphA*	*catB3*	*sul1*, *sul2*	*tetA*	*dfrA17*	*gyrA* p.S83L, *gyrA* p.D87N	*parC* p.S80I	*parE* p.S458A							*chrA*		*qacE*∆*1*
ARS-CC9092	pre-weaned calves	A	10	*aadA1*, *aph(3′)-Iia*, *aph(6)-Ic*, *aph(6)-Id*, *aph(3″)-Ib*	*bla*_TEM-1B_, *bla*_CMY-2_						*tetB*							*pcoA*, *pcoB*, *pcoC*, *pcoD*, *pcoE*, *pcoR*, *pcoS*		*silA*, *silB*, *silC*, *silP*, *silR*			*sugE1*	
ARS-CC9095	pre-weaned calves	C	88	*aph(6)-Id*, *aph(3′)-Ia*, *aph(3″)-Ib*						*sul2*	*tetB*					*ampC* promoter n.-42C>T								
ARS-CC9098	post-weaned calves	C	88	*aph(3′)-Ia*, *aph(3″)-Ib*, *aph(6)-Id*						*sul2*	*tetB*					*ampC* promoter n.-42C>T								
ARS-CC9100	post-weaned calves	B1	641	*aac(3)-Via*, *aadA24*, *aph(3″)-Ib*, *aph(3′)-Ia*, *aph(6)-Id*, *aadA5*	*bla*_TEM-1B_, *bla*_CMY-2_				*floR*	*sul1*, *sul2*	*tetM*, *tetA*	*dfrA17*											*sugE1*	*qacE*∆*1*
ARS-CC9105	dry cows	A	48	*aadA5*, *aac(3)-Via*, *aph(3′)-Ia*, *rmtE*, *aph(6)-Id*, *aadA24*, *aph(3″)-Ib*	*bla*_TEM-1B_, *bla*_CMY-2_				*floR*	*sul1*, *sul2*	*tetM*, *tetA*	*dfrA17*											*sugE1*	*qacE*∆*1*, *qacG*
ARS-CC9108	pre-weaned calves	B1	9190	*aph(3″)-Ib*, *aph(6)-Id*, *aadA2*	*bla*_CMY-2_				*floR*	*sul1*, *sul2*	*tetA*	*dfrA12*							*merA*, *merT*, *merR*, *merP*, *merC*, *merD*, *merE*			*chrA*	*sugE1*	*qacE*∆*1*
ARS-CC9117	dry cows	B1	58	*aph(3″)-Ib*, *aph(6)-Id*	*bla*_CMY-2_				*floR*	*sul2*	*tetA*													
ARS-CC9119	pre-weaned calves	B1	2522	*aph(3″)-Ib*, *aac(6′)-Iia*, *aph(6)-Id*	*bla*_CMY-2_, *bla*_TEM-1B_				*floR*	*sul1*, *sul2*	*tetA*, *tetB*								*merA*, *merT*, *merR*, *merP*, *merC*, *merD*, *merE*			*chrA*	*sugE1*	*qacE*∆*1*
ARS-CC9127	post-weaned calves	F	457	*aph(3″)-Ib*, *aph(6)-Id*	*bla*_CMY-2_				*floR*	*sul2*	*tetA*												*sugE1*	
ARS-CC9128	post-weaned calves	B1	297		*bla*_CMY-2_																		*sugE1*	
ARS-CC9129	dry cows	B1	297		*bla*_CMY-2_																		*sugE1*	
ARS-CC9131	pre-weaned calves	D	69	*aph(3′)-Ia*, *aph(6)-Id*, *aph(3″)-Ib*	*bla*_CMY-2_				*floR*	*sul2*	*tetB*						*arsA*, *arsB*, *arsC*, *arsD*							
ARS-CC9545	pre-weaned calves	A	10		*bla*_TEM-1D_						*tetA*							*pcoA*, *pcoB*, *pcoC*, *pcoD*, *pcoE*, *pcoR*, *pcoS*		*silA*, *silB*, *silC*, *silP*, *silR*				
ARS-CC9546	pre-weaned calves	B1	58	*aph(3′)-Ia*, *aph(6)-Id*, *aph(3″)-Ib*							*tetB*													
ARS-CC9550	pre-weaned calves	E	9188																					
ARS-CC9554	pre-weaned calves	B1	21	*aac(3)-Iid*, *aadA2*, *aph(6)-Id*, *aph(3″)-Ib*	*bla*_TEM-1B_			*mphA*		*sul1*, *sul2*		*dfrA12*				*ampC* promoter n.-42C>T			*merA*, *merT*, *merR*, *merP*, *merC*, *merD*, *merE*		*terW*, *terZ*, *terA*, *terB*, *terC*, *terD*, *terE*, *terF*	*chrA*		*qacE*∆*1*
ARS-CC9555	pre-weaned calves	B1	58		*bla*_CMY-2_																		*sugE1*	
ARS-CC9557	pre-weaned calves	B1	86	*aac(3)-Via*, *aph(3′)-Ia*, *aph(3″)-Ib*, *aph(6)-Id*	*bla*_CMY-2_, *bla*_TEM-1B_				*floR*	*sul1*, *sul2*	*tetA*, *tetM*		*gyrA* p.S83L	*parC* p.S80I					*merA*, *merT*, *merR*, *merP*, *merC*, *merD*, *merE*				*sugE1*	*qacE*∆*1*, *qacG*
ARS-CC9561	pre-weaned calves	B1	4086	*aph(3′)-Ia*, *aph(6)-Id*, *aph(3″)-Ib*						*sul2*	*tetB*													
ARS-CC9564	pre-weaned calves	D	106	*aadA7*, *aph(3′)-Ia*	*bla*_TEM-1B_					*sul1*	*tetA*			*parC* p.S57T			*arsA*, *arsB*, *arsC*, *arsD*		*merA*, *merT*, *merR*, *merP*, *merD*, *merE*					*qacE*∆*1*
ARS-CC9565	pre-weaned calves	A	10																					
ARS-CC9567	pre-weaned calves	E	9195								*tetA*													
ARS-CC9568	pre-weaned calves	E	5597																					
ARS-CC9570	pre-weaned calves	A	10	*aph(3″)-Ib*, *aph(3′)-Ia*, *aph(6)-Id*, *aph(4)-Ia*, *aac(3)-IV*, *aadA1*	*bla*_CMY-2_				*floR*	*sul2*	*tetB*, *tet31*, *tetA*	*dfrA1*											*sugE1*	
ARS-CC9573	pre-weaned calves	B1	17		*bla*_CTX-M-14_																*terW*, *terZ*, *terA*, *terB*, *terC*, *terD*, *terE*, *terF*			
ARS-CC9575	pre-weaned calves	A	744	*aph(3′)-Ia*, *aph(3′)-Iia*, *aadA5*, *aph(6)-Id*, *aph(3″)-Ib*	*bla*_TEM-214_			*mphA*	*floR*, *catA1*	*sul1*, *sul2*	*tetB*, *tetA*	*dfrA17*	*gyrA* p.S83L, *gyrA* p.D87N	*parC* p.A56T, *parC* p.S80I					*merA*, *merT*, *merR*, *merP*, *merD*, *merE*			*chrA*		*qacE*∆*1*
ARS-CC9705	lactating cattle	B1	58																					
ARS-CC9706	post-weaned calves	A	10	*aadA13*						*sul1*	*tetB*								*merA*, *merT*, *merR*, *merP*, *merC*, *merD*, *merE*					*qacE*∆*1*
ARS-CC9707	dry cows	A	10	*aadA13*						*sul1*	*tetB*								*merA*, *merT*, *merR*, *merP. merC*, *merD*,*merE*					*qacE*∆*1*
ARS-CC9708	pre-weaned calves	E	1131																					
ARS-CC9709	lactating cattle	B1	58																					
ARS-CC9710	post-weaned calves	A	540															*pcoA*, *pcoB*, *pcoC*, *pcoD*, *pcoE*, *pcoR*,*pcoS*		*silA*, *silB*, *silC*, *silP*, *silR*				
ARS-CC9711	lactating cattle	A	548																					
ARS-CC9712	dry cows	B1	2280																					
ARS-CC9713	pre-weaned calves	B1	765																		*terW*, *terZ*, *terA*, *terB*, *terC*, *terD*, *terE*, *terF*			
ARS-CC9714	lactating cattle	B1	8393																					
ARS-CC9715	lactating cattle	B1	4038																					
ARS-CC9716	lactating cattle	B1	109	*aadA1*						*sul1*									*merA*, *merT*, *merR*, *merP*, *merC*, *merD*, *merE*					*qacE*∆*1*
ARS-CC9717	pre-weaned calves	A	4087																					
ARS-CC9718	dry cows	E	9198																					
ARS-CC9719	dry cows	B1	164																					
ARS-CC9720	pre-weaned calves	D	2485																					
ARS-CC9721	lactating cattle	B1	603																		*terY1*, *terW*, *terZ*, *terA*, *terB*, *terC*, *terD*, *terE*, *terF*			
ARS-CC9722	lactating cattle	B1	1079																					
ARS-CC9723	pre-weaned calves	A	342				*fosA7.5*														*terW*, *terZ*, *terA*, *terB*, *terC*, *terD*, *terE*, *terF*			
ARS-CC9724	dry cows	B1	7289																					
ARS-CC9725	post-weaned calves	B1	6189																					
ARS-CC9726	lactating cattle	B1	164																					
ARS-CC9727	post-weaned calves	B1	162																					
ARS-CC9728	dry cows	B1	5221				*fosA7.5*																	
ARS-CC9730	dry cows	B1	442																					
ARS-CC9731	pre-weaned calves	F	1280								*tetB*													
ARS-CC9732	lactating cattle	B1	1727																					
ARS-CC9733	post-weaned calves	A	685																					
ARS-CC9734	lactating cattle	B1	58																					
ARS-CC9735	post-weaned calves	B1	58																					
ARS-CC9737	post-weaned calves	G	657																					
ARS-CC9738	lactating cattle	B1	1123																					
ARS-CC9739	lactating cattle	G	657																					
ARS-CC9741	lactating cattle	B1	327																					
ARS-CC9742	pre-weaned calves	B1	21																					
ARS-CC9743	lactating cattle	B1	154																					
ARS-CC9744	post-weaned calves	A	329																					
ARS-CC9745	lactating cattle	B1	847																					
ARS-CC9746	pre-weaned calves	D	137																		*terW*, *terZ*, *terA*, *terB*, *terC*, *terD*, *terE*, *terF*			
ARS-CC9747	lactating cattle	B1	1611																		*terW*, *terZ*, *terA*, *terB*, *terC*, *terD*, *terE*, *terF*			
ARS-CC9748	post-weaned calves	B1	9193	*aph(3′)-Ia*																				
ARS-CC9749	dry cows	D	6599																					
ARS-CC9750	pre-weaned calves	B1	711																					
ARS-CC9751	dry cows	C	423																					
ARS-CC9752	post-weaned calves	B1	154																					
ARS-CC9753	lactating cattle	B1	155																					
ARS-CC9755	post-weaned calves	B1	336																					
ARS-CC9756	lactating cattle	B1	278																					
ARS-CC9757	pre-weaned calves	D	32																		*terW*, *terZ*, *terA*, *terB*, *terC*, *terD*, *terE*, *terF*			
ARS-CC9758	dry cows	B1	2602																					
ARS-CC9759	post-weaned calves	A	409														*arsA*, *arsB*, *arsC*, *arsD*, *arsR*							
ARS-CC9760	post-weaned calves	C	23																					
ARS-CC9761	lactating cattle	B1	58																					
ARS-CC9762	dry cows	B1	75				*fosA7.5*																	
ARS-CC9763	dry cows	B1	164																					
ARS-CC9764	post-weaned calves	B1	1308																					
ARS-CC9765	lactating cattle	A	10																					
ARS-CC9766	lactating cattle	B1	58																					
ARS-CC9767	lactating cattle	B2	95																					
ARS-CC9768	pre-weaned calves	D	32																		*terW*, *terZ*, *terA*, *terB*, *terC*, *terD*, *terE*, *terF*			
ARS-CC9769	lactating cattle	B1	937																					
ARS-CC9770	pre-weaned calves	B1	17																		*terW*, *terZ*, *terA*, *terB*, *terC*, *terD*, *terE*, *terF*			
ARS-CC9771	dry cows	B1	1704																					
ARS-CC9772	pre-weaned calves	E	1087								*tetC*													
ARS-CC9773	lactating cattle	D	1204																					
ARS-CC9774	post-weaned calves	B1	2521																					
ARS-CC9775	dry cows	B1	711																					
ARS-CC9776	lactating cattle	B1	847																					
ARS-CC9777	post-weaned calves	B1	2521																					
ARS-CC9778	lactating cattle	D	1204								*tetC*													
ARS-CC9779	post-weaned calves	B1	392																					
ARS-CC9780	lactating cattle	A	10																					
ARS-CC9781	pre-weaned calves	B1	58																					
ARS-CC9782	lactating cattle	B1	9197																					
ARS-CC9783	post-weaned calves	B1	5730								*tetA*													
ARS-CC9784	dry cows	D	4624																					
ARS-CC9785	pre-weaned calves	A	361																					
ARS-CC9786	lactating cattle	A	10																					
ARS-CC9788	post-weaned calves	B1	711																					
ARS-CC9789	dry cows	D	38																					
ARS-CC9790	pre-weaned calves	B1	29																		*terW*, *terZ*, *terA*, *terB*, *terC*, *terD*, *terE*, *terF*			
ARS-CC9791	lactating cattle	B1	1246																					
ARS-CC9792	pre-weaned calves	B1	1308								*tetC*													
ARS-CC9793	dry cows	B1	300																		*terW*, *terZ*, *terA*, *terB*, *terC*, *terD*, *terE*, *terF*			
ARS-CC9794	post-weaned calves	B1	392																					
ARS-CC9795	lactating cattle	B1	1246																					
ARS-CC9796	lactating cattle	B1	9196				*fosA7.5*				*tetC*													
ARS-CC9798	lactating cattle	A	398								*tetC*						*arsB*, *arsD*, *arsC*, *arsD*, *asrR*	*pcoA*, *pcoB*, *pcoC*, *pcoD*, *pcoR*, *pcoS*						
ARS-CC9799	post-weaned calves	D	3509																		*terY2*, *tertY1*, *terW*, *terZ*, *terA*, *terB*, *terC*, *terD*, *terE*, *terF*			
ARS-CC9800	dry cows	B1	155																					
ARS-CC9801	pre-weaned calves	B1	22																					
ARS-CC9802	lactating cattle	B1	101																					
ARS-CC9803	pre-weaned calves	E	7244																					
ARS-CC9804	post-weaned calves	E	118																					
ARS-CC9805	lactating cattle	B1	5973																					
ARS-CC9806	post-weaned calves	B1	2521																					
ARS-CC9807	lactating cattle	B1	187																					
ARS-CC9808	dry cows	A	10															*pcoA*, *pcoB*, *pcoC*, *pcoD*, *pcoE*, *pcoR*, *pcoS*		*silA*, *silB*, *silC*, *silP*, *silR*				
ARS-CC9809	dry cows	B1	58																					
ARS-CC9810	post-weaned calves	A	329																					
ARS-CC9811	lactating cattle	A	206											*parC* p.A56T										
ARS-CC9812	post-weaned calves	B1	7812																					
ARS-CC9813	dry cows	B1	8860																					
ARS-CC9830	dry cows	E	4151																					
ARS-CC9832	lactating cattle	B1	603																					
ARS-CC9834	lactating cattle	B1	297																					
ARS-CC9835	lactating cattle	B1	58																					
ARS-CC9837	lactating cattle	B1	58																					
ARS-CC9840	lactating cattle	B1	4481																					
ARS-CC9842	dry cows	D	8651																					
ARS-CC9843	lactating cattle	B1	336																					
ARS-CC9861	pre-weaned calves	B1	2539																					
ARS-CC9862	pre-weaned calves	A	216														*arsA*, *arsB*, *arsC*, *arsD*, *arsR*	*pcoA*, *pcoB*, *pcoC*, *pcoD*, *pcoE*, *pcoR*, *pcoS*	*merA*, *merT*, *merR*, *merP*, *merC*, *merD*, *merE*	*silA*, *silB*, *silC*, *silP*, *silR*				
ARS-CC9022	pre-weaned calves	D	362	*aac(3)-Via*, *aph(3″)-Ib*, *aph(3′)-Ia*, *aph(6)-Id*	*bla*_CMY-2_, *bla*_TEM-1B_				*floR*	*sul1*, *sul2*	*tetA*, *tetB*								*merR*, *merP*, *merD*, *merE*				*sugE1*	*qacE*∆*1*, *qacG*
ARS-CC9023	pre-weaned calves	A	10	*aph(6)-Id*, *aadA1*, *aph(3′)-Ia*, *aph(3″)-Ib*	*bla*_CMY-2_, *bla*_TEM-1B_						*tetB*												*sugE1*	
ARS-CC9024	pre-weaned calves	B1	101	*aac(3)-Via*, *aph(3″)-Ib*, *aph(6)-Id*	*bla*_CMY-2_				*floR*	*sul2*	*tetA*, *tetB*												*sugE1*	
ARS-CC9025	pre-weaned calves	C	88	*aph(3′)-Ia*, *aph(6)-Id*, *aph(3″)-Ib*						*sul2*	*tetB*					*ampC* promoter n.-42C>T								
ARS-CC9026	pre-weaned calves	E	219		*bla*_CMY-2_						*tetA*												*sugE1*	
ARS-CC9027	pre-weaned calves	A	1703		*bla*_CMY-2_																		*sugE1*	
ARS-CC9028	lactating cattle	B1	515	*aph(6)-Id*, *aph(3′)-Ia*, *aph(3″)-Ib*	*bla*_TEM-1B_, *bla*_CMY-2_						*tetB*						*arsA*, *arsB*, *arsC*, *arsD*						*sugE1*	
ARS-CC9029	pre-weaned calves	C	1083	*aadA1*, *aph(3′)-Ia*	*bla*_TEM-1A_				*catA1*	*sul1*	*tetA*								*merA*, *merT*, *merR*, *merC*, *merD*, *merE*					*qacE*∆*1*
ARS-CC9032	pre-weaned calves	D	973	*aadA7*, *aph(3′)-Ia*, *aph(6)-Id*, *aph(3″)-Ib*, *aadA1*, *aadA7*	*bla*_CMY-2_, *bla*_TEM-1B_				*catA1*	*sul1*, *sul2*	*tetB*	*dfrA1*							*merA*, *merT*, *merR*, *merP*, *merC*, *merD*, *merE*				*sugE1*	*qacE*∆*1*
ARS-CC9033	lactating cattle	C	88	*aph(6)-Id*, *aph(3″)-Ib*	*bla*_CMY-2_					*sul2*		*dfrA8*												
ARS-CC9034	lactating cattle	B1	101		*bla*_TEM-1B_						*tetA*													
ARS-CC9036	post-weaned calves	D	973	*aadA7*, *aadA7*, *aph(3′)-Ia*, *aph(6)-Id*, *aph(3″)-Ib*	*bla*_CMY-2_					*sul1*, *sul2*	*tetB*								*merA*, *merT*, *merR*, *merP*, *merC*, *merD*, *merE*				*sugE1*	*qacE*∆*1*
ARS-CC9038	post-weaned calves	B1	56	*aph(3′)-Ia*, *aadA2*, *aph(6)-Id*, *aph(3″)-Ib*	*bla*_TEM-1B_					*sul1*, *sul2*	*tetA*	*dfrA12*							*merA*, *merT*, *merR*, *merP*, *merC*, *merD*, *merE*		*terY2*, *terY1*, *terW*, *terZ*, *terA*, *terB*, *terC*, *terD*, *terE*, *terF*			*qacE*∆*1*
ARS-CC9039	pre-weaned calves	A	10	*aadA2*, *aph(3′)-Ia*, *aph(3″)-Ib*, *aph(6)-Id*	*bla*_CMY-2_, *bla*_TEM-1B_			*mphA*	*floR*	*sul1*, *sul2*	*tetA*, *tetB*, *tetM*	*dfrA12*							*merT*, *merR*, *merP*, *merC*, *merD*, *merE*			*chrA*	*sugE1*	*qcG2*
ARS-CC9040	post-weaned calves	B1	6345	*aadA2*, *aph(3′)-Ia*, *aph(6)-Id*, *aph(3″)-Ib*	*bla*_CMY-2_				*floR*	*sul1*, *sul2*	*tetA*, *tetB*	*dfrA12*							*merA*, *merT*, *merR*, *merP*, *merC*, *merD*, *merE*			*chrA*	*sugE1*	*qacE*∆*1*
ARS-CC9041	pre-weaned calves	D	106	*aadA7*, *aph(3′)-Ia*	bla_TEM-1B_, *bla*_CTX-M-1_			*mphA*		*sul1*	*tetA*			*parC* p.S57T			*arsA*, *arsB*, *arsC*, *arsD*		*merA*, *merT*, *merR*, *merP*, *merC*, *merD*, *merE*					*qacE*∆*1*
ARS-CC9042	lactating cattle	B1	1252		*bla*_CTX-M-1_					*sul2*	*tetA*													
ARS-CC9043	pre-weaned calves	B1	602	*aph(3′)-Ia*, *aph(6)-Id*, *aph(3″)-Ib*	*bla*_TEM-1B_						*tetB*													
ARS-CC9044	post-weaned calves	G	117	*aadA1*, *aadA5*, *aph(3′)-Ia*	*bla*_CTX-M-14_, *bla*_TEM-1B_			*mphA*	*catA1*	*sul1*, *sul2*,	*tetA*	*dfrA17*							*merA*, *merT*, *merR*, *merP*, *merC*, *merD*, *merE*			*chrA*		*qacE*∆*1*
ARS-CC9068	pre-weaned calves	G	117	*aph(3′)-Ia*, *aadA1*, *aadA2*	*bla*_CMY-2_, *bla*_CTX-M-14_					*sul1*, *sul2*	*tetA*	*dfrA12*							*merA*, *merT*, *merR*, *merP*, *merC*, *merD*, *merE*				*sugE1*	*qacE*∆*1*
ARS-CC9046	pre-weaned calves	A	10	*aac(3)-Via*, *aadA1*, *aph(6)-Id*, *aph(3″)-Ib*, *aph(3′)-Ia*					*floR*	*sul1*, *sul2*	*tetB*, *tetA*					*ampC* promoter n.-42C>T								*qacE*∆*1*
ARS-CC9049	post-weaned calves	D	714		*bla*_CMY-2_																		*sugE1*	
ARS-CC9050	pre-weaned calves	F	967	*aph(3′)-Ia*, *aph(6)-Id*, *aph(3″)-Ib*	*bla*_CMY-2_, *bla*_TEM-1B_					*sul2*	*tetB*												*sugE1*	
ARS-CC9051	dry cows	D	106		*bla*_CMY-2_									*parC* p.S57T			*arsA*, *arsB*, *arsC*, *arsD*						*sugE1*	
ARS-CC9052	lactating cattle	D	973	*aph(3″)-Ib*, *aph(6)-Id*, *aadA1*, *aadA7*	*bla*_CMY-2_				*catA1*	*sul2*, *sul1*	*tetB*	*dfrA1*							*merA*, *merT*, *merR*, *merP*, *merC*, *merD*, *merE*		*terY2*, *terY1*, *terW*, *terZ*, *terA*, *terB*, *terC*, *terD*, *terE*, *terF*		*sugE1*	*qacE*∆*1*
ARS-CC9053	lactating cattle	C	88	*aph(3′)-Ia*, *aph(6)-Id*, *aph(3″)-Ib*						*sul2*	*tetB*					*ampC* promoter n.-42C>T								
ARS-CC9055	post-weaned calves	A	48	*aadA5*, *aph(3′)-Ia*, *aph(6)-Id*, *aph(3″)-Ib*	*bla*_CMY-2_, *bla*_TEM-1B_					*sul2*	*tetB*, *tetA*	*dfrA17*											*sugE1*	
ARS-CC9056	dry cows	A	167	*aph(3′)-Ia*, *aph(6)-Id*, *aadA5*, *aph(3″)-Ib*	*bla*_CMY-2_, *bla*_TEM-1B_				*floR*	*sul2*	*tetB*, *tetA*	*dfrA17*				*ampC* promoter n.-42C>T			*merA*, *merT*, *merR*, *merP*, *merC*, *merD*, *merE*				*sugE1*	*qacE*∆*1*
ARS-CC9057	post-weaned calves	A	167	*aph(6)-Id*, *aadA5*, *aph(3′)-Ia*, *aph(3″)-Ib*	*bla*_CMY-2_, *bla*_TEM-1B_				*floR*	*sul2*	*tetB*, *tetA*	*dfrA17*				*ampC* promoter n.-42C>T			*merA*, *merT*, *merR*, *merP*, *merC*, *merD*, *merE*				*sugE1*	*qacE*∆*1*
ARS-CC9058	lactating cattle	D	9199	*aph(6)-Id*, *aph(3″)-Ib*	*bla*_CMY-2_				*floR*	*sul2*	*tetA*								*merA*, *merT*, *merR*, *merP*, *merC*, *merD*, *merE*				*sugE1*	
ARS-CC9059	post-weaned calves	B1	17	*aph(6)-Id*, *aph(3″)-Ib*	*bla*_CMY-2_				*floR*	*sul2*	*tetA*								*merA*, *merT*, *merR*, *merP*, *merC*, *merD*, *merE*		*terW*, *terZ*, *terA*, *terB*, *terC*, *terD*, *terE*, *terF*		*sugE1*	
ARS-CC9060	pre-weaned calves	D	32	*aph(6)-Id*, *aph(3″)-Ib*	*bla*_CMY-2_					*sul2*	*tetA*								*merA*, *merT*, *merR*, *merP*, *merC*, *merD*, *merE*		*terW*, *terZ*, *terA*, *terB*, *terC*, *terD*, *terE*, *terF*		*sugE1*	
ARS-CC9061	dry cows	F	1280	*aph(3″)-Ib*, *aph(6)-Id*	*bla*_CMY-2_				*floR*	*sul2*	*tetB*, *tetA*								*merA*, *merT*, *merR*, *merP*, *merC*, *merD*, *merE*				*sugE1*	
ARS-CC9062	pre-weaned calves	A	34	*aadA1*, *aph(3′)-Ia*, *aph(6)-Id*, *aph(3″)-Ib*	*bla*_CMY-2_, *bla*_TEM-1B_					*sul2*	*tetB*	*dfrA1*											*sugE1*	
ARS-CC9063	lactating cattle	C	88	*aph(3′)-Ia*, *aph(6)-Id*, *aph(3″)-Ib*						*sul2*	*tetB*					*ampC* promoter n.-42C>T								
ARS-CC9064	pre-weaned calves	B1	940	*aadA24*, *aph(3″)-Ib*, *aph(6)-Id*	*bla*_CTX-M-14_, *bla*_OXA-1_				*catA1*	*sul2*	*tetB*	*dfrA1*						*pcoA*, *pcoB*, *pcoC*, *pcoD*, *pcoE*, *pcoR*, *pcoS*		*silA*, *silB*, *silC*, *silP*, *silR*				
ARS-CC9065	lactating cattle	B1	940	*aadA1*, *aph(3″)-Ib*, *aph(6)-Id*	bla_CTX-M-14_, *bla*_OXA-1_				*catA1*	*sul2*	*tetB*	*dfrA1*						*pcoA*, *pcoB*, *pcoC*, *pcoD*, *pcoE*, *pcoR*, *pcoS*		*silA*, *silB*, *silC*, *silP*, *silR*				

**Table 2:** Isolation source, phylogenetic group, sequence type (MLST), resistance group (MDR = multidrug-resistant; R = antimicrobial-resistant; S = antimicrobial-susceptible), plasmid replicons identified in study genomes.

Isolate ID	Sample Source	Phylogenetic Group	MLST	Resistance Group	Plasmid Replicons
ARS-CC11185	postweaned calves	G	9192	MDR	ColRNAI, IncA/C2, IncFIA, IncFIB(AP001918), IncI1_Alpha
ARS-CC11186	postweaned calves	A	1434	MDR	IncA/C2, IncX1
ARS-CC11187	preweaned calves	A	4085	MDR	ColRNAI, IncB/O/K/Z, IncFIB(AP001918), IncQ1
ARS-CC11188	postweaned calves	A	10	MDR	IncFIA, IncFIB(AP001918), IncFII, IncI1_Alpha
ARS-CC11189	lactating cattle	C	88	MDR	Col(MG828), IncFIA, IncFIB(AP001918)
ARS-CC11190	lactating cattle	B1	641	MDR	Col(MG828), Col440I, ColRNAI, IncFIB(AP001918), IncFII(pRSB107)_pRSB107, IncI1_Alpha, IncX1
ARS-CC11191	lactating cattle	C	88	R	Col156, IncI1_Alpha
ARS-CC11192	preweaned calves	A	10	MDR	Col(MG828), Col(MP18), Col156, Col440I, ColRNAI, IncFIA, IncFIB(AP001918)
ARS-CC11193	dry cows	B1	58	MDR	IncA/C2, IncFIB(AP001918)
ARS-CC11194	lactating cattle	A	10	MDR	IncA/C2
ARS-CC11195	postweaned calves	B1	9190	MDR	Col440I, ColRNAI, IncA/C2, IncFIB(AP001918), IncI1_Alpha
ARS-CC11196	dry cows	A	10	R	IncI1_Alpha, IncN
ARS-CC11197	lactating cattle	A	9189	MDR	IncA/C2
ARS-CC11198	preweaned calves	B1	58	MDR	IncFIB(AP001918), IncFII
ARS-CC11199	lactating cattle	C	88	MDR	Col(MG828), Col8282, ColRNAI, IncA/C2, IncFIB(AP001918), IncFII, IncI1_Alpha
ARS-CC11200	postweaned calves	B1	56	MDR	IncA/C2
ARS-CC11201	dry cows	D	69	R	IncFII
ARS-CC11202	lactating cattle	B1	9194	R	IncFII
ARS-CC11203	preweaned calves	B1	1049	MDR	IncFIB(AP001918), IncI1_Alpha
ARS-CC11204	postweaned calves	G	657	R	ColRNAI, IncFIB(AP001918), IncI1_Alpha
ARS-CC11205	dry cows	B1	4086	MDR	ColRNAI, IncA/C2, IncFIA, IncFIB(AP001918)
ARS-CC11206	lactating cattle	B1	9203	MDR	ColRNAI, IncFII, p0111
ARS-CC11207	postweaned calves	D	2485	R	IncFIA, IncFIB(AP001918), IncI1_Alpha
ARS-CC11208	postweaned calves	A	10	MDR	IncA/C2
ARS-CC11209	preweaned calves	A	2325	MDR	Col440II, ColRNAI, IncFIB(AP001918), IncI1_Alpha
ARS-CC11211	dry cows	B1	937	MDR	ColRNAI, IncFIB(AP001918), IncFIC(FII)
ARS-CC11212	postweaned calves	B1	1123	S	IncFIB(pB171)_pB171
ARS-CC11214	preweaned calves	B1	75	R	ColRNAI, IncFIA, IncFIB(AP001918), IncFIC(FII), p0111
ARS-CC11215	postweaned calves	B1	201	R	IncFIB(AP001918), IncI1_Alpha, IncX1
ARS-CC11216	dry cows	B1	1125	S	IncFIB(AP001918), IncFIC(FII)
ARS-CC11217	postweaned calves	B1	56	MDR	ColRNAI, IncY
ARS-CC11218	preweaned calves	D	2946	S	Col440I, IncFIB(AP001918)
ARS-CC11219	preweaned calves	C	88	MDR	IncFIA, IncFIB(AP001918)
ARS-CC11220	postweaned calves	C	9172	MDR	IncFII(pCoo)_pCoo
ARS-CC11221	dry cows	B1	6189	S	IncFIA, IncFIB(AP001918), IncFIC(FII), IncY
ARS-CC11222	preweaned calves	B1	56	MDR	IncFIB(AP001918), IncHI2A, IncHI2, RepA_pKPC-CAV1321
ARS-CC11223	postweaned calves	A	329	S	ColRNAI, IncFIB(AP001918), IncFIC(FII), IncX1, IncY
ARS-CC11224	dry cows	B1	1049	S	
ARS-CC11225	dry cows	B1	2521	S	IncFIB(AP001918), IncX1
ARS-CC11226	preweaned calves	C	23	MDR	ColRNAI, IncFIB(AP001918)
ARS-CC11227	postweaned calves	B1	1172	S	Col156, ColRNAI, IncFIB(AP001918), IncFIC(FII), IncX1, IncX3
ARS-CC11228	lactating cattle	B1	101	MDR	IncFIA(HI1)_HI1, IncFIB(pB171)_pB171
ARS-CC11229	preweaned calves	B2	4260	S	IncFIB(AP001918)
ARS-CC11230	dry cows	A	8935	S	ColRNAI, IncFIC(FII), IncI1_Alpha
ARS-CC11231	lactating cattle	A	1101	MDR	ColRNAI, IncFIB(AP001918), IncFIC(FII), IncFII
ARS-CC11232	lactating cattle	B1	56	MDR	
ARS-CC11233	preweaned calves	B1	58	MDR	ColRNAI, IncI2_Delta
ARS-CC11234	postweaned calves	E	1140	MDR	ColRNAI, IncA/C2
ARS-CC11235	dry cows	B2	95	R	ColRNAI, IncFIB(AP001918), IncX1
ARS-CC11236	lactating cattle	B1	937	MDR	IncFIB(AP001918), IncFIC(FII), IncI1_Alpha
ARS-CC11237	preweaned calves	D	973	MDR	IncFIA, IncFIB(AP001918)
ARS-CC11238	lactating cattle	B1	442	MDR	IncFIA, IncFIB(AP001918), IncX1
ARS-CC11239	postweaned calves	A	329	MDR	IncFIB(AP001918)
ARS-CC11240	postweaned calves	B1	101	MDR	Col440I, ColRNAI, IncFIB(AP001918), IncFII(pHN7A8)_pHN7A8
ARS-CC11241	preweaned calves	A	10	MDR	IncFIA, IncFIB(AP001918)
ARS-CC11242	postweaned calves	B1	2522	MDR	Col156
ARS-CC11243	preweaned calves	E	57	MDR	IncA/C2
ARS-CC11244	postweaned calves	B1	446	MDR	Col440I, IncFIB(AP001918)
ARS-CC11245	dry cows	B1	56	MDR	
ARS-CC11246	postweaned calves	B1	1844	S	
ARS-CC11247	post-weaned calves	B1	278	MDR	Col440I, ColRNAI
ARS-CC11248	lactating cattle	B1	58	MDR	Col8282, ColRNAI, ColpVC
ARS-CC11249	preweaned calves	A	10	MDR	IncFIA, IncFIB(AP001918), IncFII, IncI1_Alpha
ARS-CC11250	preweaned calves	A	9191	MDR	Col440I, IncFIA, IncFIB(pB171)_pB171, IncI_Gamma, IncX1
ARS-CC11251	preweaned calves	A	93	MDR	Col156, ColRNAI, IncB/O/K/Z, IncFIA, IncFIB(AP001918)
ARS-CC11252	postweaned calves	A	744	MDR	IncFIA, IncFIB(AP001918), IncFII(pAMA1167-NDM-5)_pAMA1167-NDM-5
ARS-CC11253	preweaned calves	C	23	MDR	ColRNAI, IncFIB(AP001918)
ARS-CC11254	postweaned calves	B1	58	MDR	IncFIB(AP001918)
ARS-CC11255	postweaned calves	A	206	MDR	Col156, IncA/C2, IncFII(pSE11)_pSE11, IncHI2A, IncHI2, RepA_pKPC-CAV1321, p0111
ARS-CC11256	postweaned calves	B1	155	MDR	IncFIB(AP001918)
ARS-CC11257	preweaned calves	G	657	S	ColRNAI, IncY
ARS-CC11258	lactating cattle	B1	164	MDR	Col440I, ColRNAI, IncFIA, IncFIB(AP001918), IncI2_Delta
ARS-CC11260	postweaned calves	B1	155	S	IncFIB(AP001918), IncI1_Alpha
ARS-CC11261	dry cows	E	4175	S	ColRNAI, IncFIA, IncFIB(AP001918)
ARS-CC11262	dry cows	B1	2163	S	
ARS-CC11263	dry cows	B1	278	MDR	ColRNAI, IncFIB(AP001918), IncFIC(FII)
ARS-CC11264	preweaned calves	B1	8185	S	IncFIA, IncFIB(AP001918), IncX1
ARS-CC11265	dry cows	B1	13	S	IncFIA, IncFIB(AP001918), IncFIC(FII)
ARS-CC11266	dry cows	B1	4481	S	IncFIA, IncFIB(AP001918)
ARS-CC11267	preweaned calves	B1	21	MDR	IncA/C2, IncB/O/K/Z, IncFIB(AP001918), IncY
ARS-CC11268	postweaned calves	A	6927	MDR	Col440I, IncA/C2, IncFIA, IncFIB(pB171)_pB171
ARS-CC11269	postweaned calves	B2	95	R	IncFIB(AP001918), IncX1, IncY
ARS-CC11270	dry cows	B1	56	S	Col(MG828), IncFIA, IncFIB(AP001918), IncFIC(FII), IncI1_Alpha
ARS-CC11271	preweaned calves	B1	6559	S	
ARS-CC11272	dry cows	G	9192	MDR	ColRNAI, IncFIA, IncFIB(AP001918), IncI1_Alpha
ARS-CC11273	lactating cattle	G	9192	MDR	ColRNAI, IncFIA, IncFIB(AP001918), IncI1_Alpha
ARS-CC11274	lactating cattle	B1	316	MDR	ColRNAI, IncFIB(AP001918)
ARS-CC7050	lactating cattle	A	617	MDR	ColRNAI, IncFIA, IncFIB(AP001918)
ARS-CC9092	preweaned calves	A	10	MDR	IncFII, IncR
ARS-CC9095	preweaned calves	C	88	MDR	IncFIA, IncFIB(AP001918)
ARS-CC9098	postweaned calves	C	88	MDR	IncFIA, IncFIB(AP001918)
ARS-CC9100	postweaned calves	B1	641	MDR	IncA/C2, IncX1, IncX3, IncX4, IncY
ARS-CC9105	dry cows	A	48	MDR	IncA/C2
ARS-CC9108	preweaned calves	B1	9190	MDR	Col440I, ColRNAI, IncA/C2, IncFIB(AP001918)
ARS-CC9117	dry cows	B1	58	MDR	IncFIB(AP001918), IncY
ARS-CC9119	preweaned calves	B1	2522	MDR	IncA/C2
ARS-CC9127	postweaned calves	F	457	MDR	ColRNAI, IncFII, IncI2_Delta
ARS-CC9128	postweaned calves	B1	297	R	ColRNAI, IncA/C2, IncI1_Alpha
ARS-CC9129	dry cows	B1	297	R	ColRNAI, IncI1_Alpha
ARS-CC9131	preweaned calves	D	69	MDR	ColRNAI, IncFIA, IncFIB(AP001918), IncI1_Alpha
ARS-CC9545	preweaned calves	A	10	R	Col156, IncFIA, IncFIB(AP001918), IncI2_Delta, IncX3
ARS-CC9546	preweaned calves	B1	58	R	IncFIA(HI1)_HI1, IncFIB(pB171)_pB171
ARS-CC9550	preweaned calves	E	9188	S	IncI1_Alpha
ARS-CC9554	preweaned calves	B1	21	MDR	Col(MG828), ColRNAI, IncB/O/K/Z, IncFIB(AP001918), p0111
ARS-CC9555	preweaned calves	B1	58	R	IncFIA(HI1)_HI1, IncFIB(pB171)_pB171, IncI1_Alpha
ARS-CC9557	preweaned calves	B1	86	MDR	IncA/C2, IncY
ARS-CC9561	preweaned calves	B1	4086	MDR	ColRNAI, IncI2_Delta
ARS-CC9564	preweaned calves	D	106	MDR	IncFIA, IncFIB(AP001918)
ARS-CC9565	preweaned calves	A	10	S	ColRNAI, IncFIB(pB171)_pB171
ARS-CC9567	preweaned calves	E	9195	R	IncFIB(AP001918)
ARS-CC9568	preweaned calves	E	5597	S	IncFIB(AP001918)
ARS-CC9570	preweaned calves	A	10	MDR	Col(MG828), Col440I, ColRNAI, IncA/C2, IncFIA, IncFIB(AP001918)
ARS-CC9573	preweaned calves	B1	17	R	ColRNAI, IncFIB(AP001918), IncFII
ARS-CC9575	preweaned calves	A	744	MDR	ColRNAI, IncX1
ARS-CC9705	lactating cattle	B1	58	S	IncFIB(AP001918), IncFII(pHN7A8)_pHN7A8
ARS-CC9706	postweaned calves	A	10	MDR	ColRNAI, IncFIA, IncFIB(AP001918), IncFII(pHN7A8)_pHN7A8, IncFII
ARS-CC9707	dry cows	A	10	MDR	ColRNAI, IncFIA, IncFIB(AP001918), IncFII(pHN7A8)_pHN7A8, IncFII
ARS-CC9708	preweaned calves	E	1131	S	Col440I
ARS-CC9709	lactating cattle	B1	58	S	IncFIA, IncFIB(AP001918)
ARS-CC9710	postweaned calves	A	540	S	
ARS-CC9711	lactating cattle	A	548	S	ColRNAI, IncFIB(AP001918)
ARS-CC9712	dry cows	B1	2280	S	
ARS-CC9713	preweaned calves	B1	765	S	IncFIC(FII)
ARS-CC9714	lactating cattle	B1	8393	S	
ARS-CC9715	lactating cattle	B1	4038	S	IncFIA, IncFIB(AP001918), IncFIC(FII)
ARS-CC9716	lactating cattle	B1	109	R	
ARS-CC9717	preweaned calves	A	4087	S	IncFIA, IncFIB(AP001918)
ARS-CC9718	dry cows	E	9198	S	IncFIA, IncFIB(AP001918), IncFIC(FII)
ARS-CC9719	dry cows	B1	164	S	IncFIA, IncFIB(AP001918), IncFIC(FII)
ARS-CC9720	preweaned calves	D	2485	S	Col440I, ColRNAI, IncFIA, IncFIB(pB171)_pB171
ARS-CC9721	lactating cattle	B1	603	S	ColRNAI, IncFIA, IncFIB(AP001918)
ARS-CC9722	lactating cattle	B1	1079	S	ColRNAI
ARS-CC9723	preweaned calves	A	342	R	Col(MG828), Col156, ColRNAI, IncFIB(AP001918)
ARS-CC9724	dry cows	B1	7289	S	ColRNAI, IncFIA, IncFIB(AP001918)
ARS-CC9725	postweaned calves	B1	6189	S	IncFIA, IncFIB(AP001918), IncFIC(FII), IncY
ARS-CC9726	lactating cattle	B1	164	S	IncFIA
ARS-CC9727	postweaned calves	B1	162	S	ColRNAI, IncFIB(AP001918)
ARS-CC9728	dry cows	B1	5221	R	IncFIA, IncFIB(AP001918), IncFIC(FII), IncY
ARS-CC9730	dry cows	B1	442	S	IncFIA, IncFIB(AP001918)
ARS-CC9731	preweaned calves	F	1280	R	ColRNAI
ARS-CC9732	lactating cattle	B1	1727	S	IncFIB(AP001918)
ARS-CC9733	postweaned calves	A	685	S	IncI1_Alpha
ARS-CC9734	lactating cattle	B1	58	S	IncFIA, IncFIB(AP001918), IncFIC(FII)
ARS-CC9735	postweaned calves	B1	58	S	IncFIB(AP001918)
ARS-CC9737	postweaned calves	G	657	S	IncFIB(AP001918)
ARS-CC9738	lactating cattle	B1	1123	S	IncFIA(HI1)_HI1, IncFIB(K)_Kpn3
ARS-CC9739	lactating cattle	G	657	S	Col(MG828), IncB/O/K/Z, IncFIB(AP001918)
ARS-CC9741	lactating cattle	B1	327	S	IncFIB(AP001918)
ARS-CC9742	preweaned calves	B1	21	S	Col440I, IncB/O/K/Z, IncFIB(AP001918)
ARS-CC9743	lactating cattle	B1	154	S	IncFIA, IncFIC(FII)
ARS-CC9744	postweaned calves	A	329	S	ColRNAI, IncFIB(AP001918), IncX1, IncY
ARS-CC9745	lactating cattle	B1	847	S	Col(MG828), ColRNAI, ColpVC, IncI1_Alpha
ARS-CC9746	preweaned calves	D	137	S	IncFIB(AP001918), IncY
ARS-CC9747	lactating cattle	B1	1611	S	IncFIB(AP001918), IncFII(pHN7A8)_pHN7A8
ARS-CC9748	postweaned calves	B1	9193	R	ColRNAI, IncFIB(AP001918)
ARS-CC9749	dry cows	D	6599	S	IncI1_Alpha
ARS-CC9750	preweaned calves	B1	711	S	IncFIB(AP001918)
ARS-CC9751	dry cows	C	423	S	ColRNAI, IncFIA, IncFIB(AP001918)
ARS-CC9752	postweaned calves	B1	154	S	IncFIA, IncFIB(AP001918)
ARS-CC9753	lactating cattle	B1	155	S	IncFIB(AP001918), IncI1_Alpha, IncX4
ARS-CC9755	postweaned calves	B1	336	S	IncFIB(AP001918), IncFIC(FII)
ARS-CC9756	lactating cattle	B1	278	S	IncFIB(AP001918), IncFIC(FII), IncFII, IncI1_Alpha
ARS-CC9757	preweaned calves	D	32	S	IncFIB(AP001918)
ARS-CC9758	dry cows	B1	2602	S	ColRNAI, IncFIB(AP001918), IncI2_Delta, IncY
ARS-CC9759	postweaned calves	A	409	S	IncFIB(K)_Kpn3, IncY
ARS-CC9760	postweaned calves	C	23	S	Col440I, IncFIA, IncFIB(pB171)_pB171
ARS-CC9761	lactating cattle	B1	58	S	IncFIB(AP001918), IncFIC(FII), IncX1
ARS-CC9762	dry cows	B1	75	R	ColRNAI, IncFIA, IncFIB(AP001918), IncFIC(FII), IncFII(pCoo)_pCoo,
ARS-CC9763	dry cows	B1	164	S	IncFIA, IncFIB(AP001918)
ARS-CC9764	postweaned calves	B1	1308	S	IncFIB(AP001918)
ARS-CC9765	lactating cattle	A	10	S	
ARS-CC9766	lactating cattle	B1	58	S	Col440I, IncI1_Alpha, IncI2_Delta
ARS-CC9767	lactating cattle	B2	95	R	IncFIB(AP001918), IncX1
ARS-CC9768	preweaned calves	D	32	S	IncB/O/K/Z, IncFIB(AP001918)
ARS-CC9769	lactating cattle	B1	937	S	
ARS-CC9770	preweaned calves	B1	17	S	IncFIB(AP001918)
ARS-CC9771	dry cows	B1	1704	S	ColRNAI, IncFIB(AP001918), IncI1_Alpha
ARS-CC9772	preweaned calves	E	1087	R	IncFIB(AP001918), IncY
ARS-CC9773	lactating cattle	D	1204	S	ColRNAI, IncFIB(AP001918)
ARS-CC9774	postweaned calves	B1	2521	S	Col440I, IncFIA, IncFIB(AP001918), IncX1, IncX4
ARS-CC9775	dry cows	B1	711	S	IncFIA, IncFIB(AP001918), IncFIC(FII)
ARS-CC9776	lactating cattle	B1	847	S	Col440II, ColRNAI, ColpVC, IncI1_Alpha, p0111
ARS-CC9777	postweaned calves	B1	2521	S	ColRNAI, IncFIB(AP001918)
ARS-CC9778	lactating cattle	D	1204	R	IncFIB(AP001918), IncI2_Delta, IncY
ARS-CC9779	postweaned calves	B1	392	S	IncFIA(HI1)_HI1, IncFIB(AP001918), IncFIC(FII)
ARS-CC9780	lactating cattle	A	10	S	ColRNAI, IncFIC(FII)
ARS-CC9781	preweaned calves	B1	58	S	IncFIA(HI1)_HI1, IncFIB(pB171)_pB171, IncY
ARS-CC9782	lactating cattle	B1	9197	S	Col440I, ColRNAI, IncFIA, IncFIB(AP001918), IncFIC(FII), IncI2_Delta
ARS-CC9783	postweaned calves	B1	5730	R	Col(MG828), Col440I, Col8282, ColRNAI, IncFII, IncN
ARS-CC9784	dry cows	D	4624	S	Col156, ColRNAI, IncFIA, IncFIB(AP001918), IncFIC(FII), IncI2_Delta
ARS-CC9785	preweaned calves	A	361	S	
ARS-CC9786	lactating cattle	A	10	S	ColRNAI, IncFIB(pB171)_pB171
ARS-CC9788	postweaned calves	B1	711	S	ColRNAI, IncFIB(AP001918), IncI1_Alpha
ARS-CC9789	dry cows	D	38	S	IncFIA, IncFIB(AP001918), IncFIC(FII)
ARS-CC9790	preweaned calves	B1	29	S	ColRNAI, IncFIB(AP001918)
ARS-CC9791	lactating cattle	B1	1246	S	ColRNAI, IncFIA, IncI1_Alpha
ARS-CC9792	preweaned calves	B1	1308	R	IncFIB(pB171)_pB171
ARS-CC9793	dry cows	B1	300	S	Col156, IncFIB(AP001918)
ARS-CC9794	postweaned calves	B1	392	S	IncFIA(HI1)_HI1, IncFIB(AP001918), IncFIC(FII), IncI1_Alpha
ARS-CC9795	lactating cattle	B1	1246	S	ColRNAI, IncFIA, IncI1_Alpha
ARS-CC9796	lactating cattle	B1	9196	R	IncFIB(AP001918), pENTAS02
ARS-CC9798	lactating cattle	A	398	R	IncFIA, IncFII
ARS-CC9799	postweaned calves	D	3509	S	ColpVC, IncA/C2, IncFII(pCoo)_pCoo, IncHI2A, IncHI2, RepA_pKPC-CAV1321
ARS-CC9800	dry cows	B1	155	S	ColRNAI
ARS-CC9801	preweaned calves	B1	22	S	
ARS-CC9802	lactating cattle	B1	101	S	IncFIA, IncFIB(AP001918)
ARS-CC9803	preweaned calves	E	7244	S	IncFIB(AP001918)
ARS-CC9804	postweaned calves	E	118	S	ColRNAI, IncFIB(AP001918)
ARS-CC9805	lactating cattle	B1	5973	S	ColRNAI, IncFIA, IncFIB(AP001918)
ARS-CC9806	postweaned calves	B1	2521	S	IncFIA(HI1)_HI1, IncFIB(pB171)_pB171
ARS-CC9807	lactating cattle	B1	187	S	IncFIA, IncFIB(AP001918), IncY
ARS-CC9808	dry cows	A	10	S	Col440I
ARS-CC9809	dry cows	B1	58	S	IncFIA, IncFIB(AP001918)
ARS-CC9810	postweaned calves	A	329	S	ColRNAI, IncFIB(AP001918), IncI2_Delta
ARS-CC9811	lactating cattle	A	206	R	
ARS-CC9812	postweaned calves	B1	7812	S	ColRNAI, IncFIB(AP001918), IncY
ARS-CC9813	dry cows	B1	8860	S	IncFIA, IncFIB(AP001918), IncFIC(FII)
ARS-CC9830	dry cows	E	4151	S	IncFIA, IncFIB(AP001918)
ARS-CC9832	lactating cattle	B1	603	S	Col156, Col440I, ColRNAI, IncFIB(AP001918), IncFIC(FII), IncI2_Delta
ARS-CC9834	lactating cattle	B1	297	S	IncFIA, IncFIB(AP001918)
ARS-CC9835	lactating cattle	B1	58	S	ColRNAI, IncFIA, IncFIB(AP001918), IncFIC(FII), IncI_Gamma
ARS-CC9837	lactating cattle	B1	58	S	Col440I, IncY
ARS-CC9840	lactating cattle	B1	4481	S	IncFIA, IncFIB(AP001918)
ARS-CC9842	dry cows	D	8651	S	IncY
ARS-CC9843	lactating cattle	B1	336	S	IncFIA, IncFIB(AP001918)
ARS-CC9861	preweaned calves	B1	2539	S	IncFIA, IncFIB(AP001918), IncI1_Alpha
ARS-CC9862	preweaned calves	A	216	S	Col440II, Col440I, IncFIA(HI1)_HI1, IncFIB(K)_Kpn3, IncHI1A, IncHI1B(R27)_R27
ARS-CC9022	preweaned calves	D	362	MDR	Col156, ColRNAI, IncA/C2, IncFIA, IncFIB(AP001918)
ARS-CC9023	preweaned calves	A	10	MDR	IncFIB(AP001918), IncI1_Alpha
ARS-CC9024	preweaned calves	B1	101	MDR	IncA/C2, IncFIA(HI1)_HI1, IncFIB(pB171)_pB171
ARS-CC9025	preweaned calves	C	88	MDR	ColRNAI, IncFIA, IncFIB(AP001918)
ARS-CC9026	preweaned calves	E	219	R	IncY
ARS-CC9027	preweaned calves	A	1703	R	IncFII, IncQ1
ARS-CC9028	lactating cattle	B1	515	MDR	IncFIB(AP001918), IncI1_Alpha
ARS-CC9029	preweaned calves	C	1083	MDR	IncFIB(AP001918), IncI2_Delta
ARS-CC9032	preweaned calves	D	973	MDR	IncB/O/K/Z, IncFIA, IncFIB(AP001918)
ARS-CC9033	lactating cattle	C	88	MDR	ColRNAI, IncFII, IncI1_Alpha
ARS-CC9034	lactating cattle	B1	101	R	IncFIB(AP001918), IncFIC(FII), IncX1, IncX3
ARS-CC9036	postweaned calves	D	973	MDR	IncFIA, IncFIB(AP001918)
ARS-CC9038	postweaned calves	B1	56	MDR	IncFIB(AP001918), IncHI2A, IncHI2, RepA_pKPC-CAV1321
ARS-CC9039	preweaned calves	A	10	MDR	IncA/C2, IncFIB(AP001918), IncI2_Delta, IncX1
ARS-CC9040	postweaned calves	B1	6345	MDR	IncY
ARS-CC9041	preweaned calves	D	106	MDR	IncFIA, IncFIB(AP001918), IncI1_Alpha, IncN
ARS-CC9042	lactating cattle	B1	1252	MDR	IncFIB(AP001918), IncFIC(FII), IncI1_Alpha
ARS-CC9043	preweaned calves	B1	602	MDR	IncFIB(AP001918), IncY
ARS-CC9044	postweaned calves	G	117	MDR	Col(MG828), Col440I, ColRNAI, IncFIA, IncFIB(AP001918), IncFII
ARS-CC9068	preweaned calves	G	117	MDR	Col8282, ColRNAI, IncFIA, IncFIB(AP001918), IncFII
ARS-CC9046	preweaned calves	A	10	MDR	IncFIA, IncFIB(AP001918), IncI1_Alpha
ARS-CC9049	postweaned calves	D	714	R	IncFIB(AP001918), IncFII, IncI2_Delta
ARS-CC9050	preweaned calves	F	967	MDR	IncFIA, IncFIB(AP001918), IncI1_Alpha, IncI2_Delta
ARS-CC9051	dry cows	D	106	R	ColRNAI, IncI1_Alpha
ARS-CC9052	lactating cattle	D	973	MDR	IncFIA, IncFIB(AP001918), IncHI2A, IncHI2, IncI1_Alpha, RepA_pKPC-CAV1321
ARS-CC9053	lactating cattle	C	88	MDR	IncFIA, IncFIB(AP001918)
ARS-CC9055	postweaned calves	A	48	MDR	IncFIB(AP001918), IncFIC(FII), IncI1_Alpha, IncR, IncY
ARS-CC9056	dry cows	A	167	MDR	Col(MG828), Col156, ColRNAI, IncA/C2, IncFIA, IncFIB(AP001918), IncY
ARS-CC9057	postweaned calves	A	167	MDR	Col(MG828), Col156, ColRNAI, IncA/C2, IncFIA, IncFIB(AP001918), IncY
ARS-CC9058	lactating cattle	D	9199	MDR	IncA/C2
ARS-CC9059	postweaned calves	B1	17	MDR	Col(MG828), IncA/C2, IncFIB(AP001918)
ARS-CC9060	preweaned calves	D	32	MDR	IncA/C2, IncB/O/K/Z, IncFIB(AP001918)
ARS-CC9061	dry cows	F	1280	MDR	ColRNAI, IncA/C2
ARS-CC9062	preweaned calves	A	34	MDR	Col(MG828), IncFIA, IncFIB(AP001918)
ARS-CC9063	lactating cattle	C	88	MDR	IncFIA, IncFIB(AP001918), IncX4
ARS-CC9064	preweaned calves	B1	940	MDR	ColE10, ColRNAI, IncFII
ARS-CC9065	lactating cattle	B1	940	MDR	ColE10, ColRNAI, IncFII

**Table 3:** Operons of interest that were enriched in MDR genomes. Also listed are the functions of these operons, the ranges of percent presence in MDR strains (not all genes within an operon were detected), maximum percent presence in susceptible strains, and the q-values indicating the statistical significance of their enrichment in MDR genomes (Fisher’s Exact Test).

Operon	Function	Carried by MDR Strains (%)	Carried by Susceptible Strains (%)	q-Values
*iucABCD-iutA*	Aerobactin synthesis/receptor	48%	6%	1.61 × 10^−9^
*sitACD*	Iron ABC transporter	28 to 42%	8%	1.71 × 10^−6^
*papABCDEGHIJK*	P fimbriae	30% to 53%	11%	7.99 × 10^−5^
*fecABCDE*	Ferric citrate transport system	60 to 62%	24%	0.00016
*iolABCDEG-iatA*	*myo*-inositol transport and utilization	22%	3%	0.0023
*alsABC*	D-allose transport system	49%	21%	0.0087
*ulaABC*	Ascorbate transport	50 to 56%	26%	0.013

## Data Availability

All genome sequencing data generated in this study are publicly available at NCBI (Supplementary File S4).

## Supplementary Materials

The following supporting information can be downloaded at https://www.mdpi.com/article/10.3390/antibiotics12101559/s1, Supplementary File S1: Co-occurrence results between antimicrobial-resistance genes (ARGs), biocide resistance genes (BRGs), and metal resistance genes (MRGs). Supplementary File S2: Full virulence matrix of all study strains is used in this analysis. Supplementary File S3: Genes enriched in MDR and susceptible genes along with their corresponding *E. coli* NCBI protein ID, BRG/MRG, annotation, SignalP identity, and KEGG orthology (KO). Supplementary File S4: NCBI accession numbers of *E. coli* genomes sequenced for this study.
